# Ultra-efficient, Anisotropic Cellulose Aerogel with Polydopamine Interfacial Bridged Structure and Photothermal Modification for Seawater Desalination

**DOI:** 10.34133/research.0888

**Published:** 2025-09-25

**Authors:** Miao Sun, Xin Wang, Yuan Yu, Meichen Li, Meng Wang, Wenbo Zhang, Zhaolin Yang, Jiazuo Zhou, Haiyue Yang, Chengyu Wang

**Affiliations:** ^1^ Key Laboratory of Bio-Based Material Science and Technology of Ministry of Education, Northeast Forestry University, Harbin 150040, PR China.; ^2^ State Key Laboratory of Urban Water Resource and Environment, School of Environment, Harbin Institute of Technology, Harbin 150001, PR China.

## Abstract

Solar-driven interfacial evaporators represent a promising technique to address the energy crisis and freshwater scarcity issues. However, simultaneously achieving both high evaporation efficiency and long-term stability in a single evaporator system under multifactorial environmental conditions remains challenging. Herein, inspired by the anisotropic channel structure of wood, a novel evaporator featuring a vertical channel structure and excellent photothermal conversion performance for evaporation is developed through the covalent-bond bridging of MXene–polydopamine–cellulose nanocrystals. Polydopamine-modified MXene acts as a photothermal material, exhibiting excellent photothermal conversion efficiency. The vertical channels endow the evaporator with efficient thermal management and rapid mass transfer capabilities to dynamically balance the feedwater supply and photothermic energy input. Thereby, the evaporator exhibits an enhanced evaporation rate of 2.29 kg m^−2^ h^−1^, accompanied by a remarkable 97.34% evaporation efficiency under 1-sun illumination. Additionally, the evaporator possesses a mechanical strength as high as 0.454 MPa, which ensures its long-term stability. Over 14 d of testing, stable evaporation rates of 2.27 kg m^−2^ h^−1^ were maintained over 8-h cycles with no salt crystallization. This work introduces a novel evaporator design with a strong evaporation capability, which has potential applications in addressing the energy crisis and water scarcity challenges.

## Introduction

Industrial progress fuels ever-growing demands for conventional energy sources. The consequent energy crisis has exacerbated the global freshwater scarcity, bringing substantial challenges to the sustainable advancement of human society [1]. Seawater desalination has attracted increasing attention as a proven and scalable strategy to obtain fresh water and has been recognized as a crucial method for combating international freshwater scarcity [2,3]. Among emerging desalination methods, the solar-driven interfacial evaporator has drawn considerable attention for its outstanding photothermal conversion capability, minimal energy consumption, operational safety, and eco-compatibility [4,5]. To translate these advantages into practical applications, developing evaporation systems with high efficiency and long-term stability under multifactorial environments is imperative for advancing evaporator technologies [6,7].

Developing high-efficiency and long-term-stability evaporators fundamentally depends on the synergistic integration of 4 critical components: (a) efficient photothermal conversion, (b) good thermal insulation, (c) reduced evaporation enthalpy, and (d) rapid water transport capability [8]. Important resources have been focused on developing innovative photothermal materials to optimize solar–thermal energy conversion [9–14]. Among these, 2-dimensional MXene stands out as particularly promising due to its superior surface-to-volume ratio, good mechanical flexibility, and high charge carrier mobility [15]. While its localized surface plasmon resonance (LSPR) effect [16] achieves high visible (Vis)-region light capture, its weak near-infrared (NIR) light absorption reduces its photothermal efficiency across the full spectrum [17]. Charge-transfer engineering of MXene’s surface Ti electronic structure can enhance NIR absorption and nonradiative relaxation, synergizing with LSPR to broaden the Vis–NIR range and improve photothermal conversion [18–20]. As a π-conjugated polymer exhibiting broadband Vis–NIR absorption and exceptional interfacial adhesion [21], polydopamine (PDA) can be used as an ideal modifier to optimize the electronic structure of nanocomposites through multiple interactions [22], including van der Waals forces, π–π conjugation, and hydrogen bonding [23]. PDA-modified MXene (PDMM) not only exhibits a broadened Vis–NIR absorption range but also demonstrates an enhanced LSPR effect. [24]. However, its evaporation performance remains constrained by its inherent limitations in thermal insulation and evaporation enthalpy.

The strategic integration of cellulose nanocrystals (CNCs) into PDMM offers a promising solution to overcome these limitations. The intrinsically low thermal conductivity of CNCs establishes an insulating network within the MXene–PDA–CNC composite, while abundant hydrophilic groups (–OH and –NH) from both PDA and CNCs form a hydrated polymer network through hydrogen bonding [25,26]. This network not only facilitates the generation of low-enthalpy active water but also minimizes the activation energy for phase change, thus optimizing the evaporation efficiency [27]. However, the multimechanistic synergistic bridging effects of the MXene–PDA–CNC system, particularly its structure–property relationships, have not yet been systematically investigated. Moreover, despite enhanced evaporation through interfacial engineering, salt accumulation and rate limitations restrict practical desalination applications. To address these challenges, incorporating a 3-dimensional (3D) structure proves advantageous for simultaneously managing water transport and preventing salt crystallization [28].

Inspired by the transpiration process of wood, where water is efficiently transported vertically [29], a 3D evaporator featuring oriented channels can break the conventional evaporation limit of 1.47 kg m^−2^ h^−1^ achieved for 2-dimensional systems [30]. The oriented channel structure enhances capillary action and expands vapor exchange surfaces, significantly boosting evaporation efficiency [31]. Additionally, the proposed structural design addresses the salt accumulation issue that plagues conventional random networks. The oriented channel architecture enables the occurrence of 3 synergistic mechanisms: (a) rapid water transport, (b) enhanced reverse diffusion of salt ions, and (c) continuous convective outflow remaining in subsaturation conditions [32,33]. This guarantees durable performance of the evaporation system under practical working conditions. While such innovative structure addresses the abovementioned fundamental limitations, systematic studies combining bridging strategies with an oriented channel structure for desalination applications are still lacking.

Herein, a high-efficiency and long-term-stability evaporator integrating an oriented channel structure with a PDA interfacial bridging strategy was successfully constructed for the first time via the freeze-casting method [34–36]. PDA acts as a multifunctional bridge, which boosts the photothermal activity of MXene through surface modification and simultaneously forms covalent cross-links with CNCs. By simultaneously achieving a reduction in thermal conductivity (*κ* = 0.01 W m^−1^ K^−1^) and a decrease in enthalpy of evaporation (Δ*H* = 1,761 kJ kg^−1^), this bridging approach enables an exceptional 2.29 kg m^−2^ h^−1^ evaporation rate under 1-sun illumination. The structure–property relationship of the MXene–PDA–CNC network was systematically investigated through density functional theory (DFT) calculations. Furthermore, the oriented channel structure enables sufficient water replenishment, thus avoiding salt crystallization congestion, allowing the evaporator to operate steadily for 14 consecutive days with excellent evaporation performance and stability. This work provides guidance for achieving evaporators with high efficiency and stable performance by coupling photothermal conversion, thermal insulation, reduced evaporation enthalpy, and rapid water transport capability. It is envisaged that these results will facilitate the design of highly efficient systems to address the issue of freshwater scarcity.

## Results

### Synthesis process of the ACPMA evaporator

**Fig. 1.**
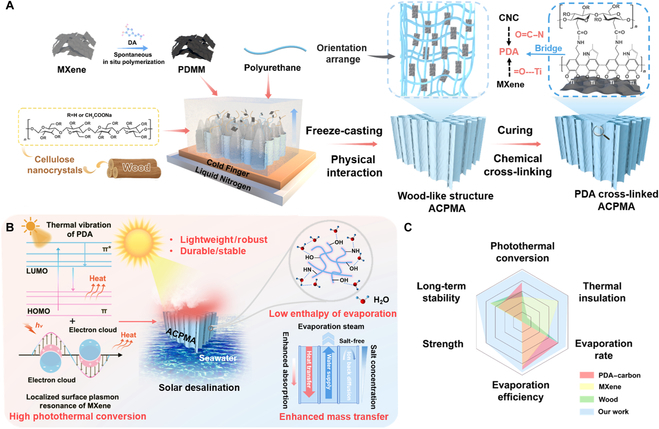
Schematic of the anisotropic cellulose nanocrystal/polydopamine-modified MXene aerogel (ACPMA) evaporator fabrication protocol. (A) Scheme for preparation process of the ACPMA evaporator featuring an oriented channel structure for solar desalination. (B) A diagram illustration of ACPMA’s high evaporation performance, indicating the coupling of photothermal conversion, microstructure, water supply, and vaporization enthalpy. (C) Illustration of the prominent advantages compared to other evaporators. DA, dopamine; PDMM, polydopamine-modified MXene; CNC, cellulose nanocrystal; PDA, polydopamine; LUMO, lowest unoccupied molecular orbital; HOMO, highest occupied molecular orbital.

To obtain an evaporator combining superior thermal management performance and enhanced mechanical stability, an anisotropic CNC/PDMM aerogel (ACPMA) evaporator was engineered to achieve exceptional efficiency (Fig. 1A). MXene nanosheets with surface-terminated –OH and –Ti groups serve as nucleation centers for PDA deposition [37]. Multiple reactive groups in PDA, including catechol, hydroxyl, and amino groups, establish robust molecular linkages with MXene through dual bonding mechanisms combining covalent bonds and hydrogen bonds, thus forming PDMM nanosheets. PDA not only modifies MXene but also acts as a bridge for denser cross-linking with the CNCs, which increases the interfacial force between the components. Furthermore, the synergistic effect of the thermal vibration of PDA and the LSPR effect of MXene leads to an exceptional photothermal performance of the PDMM nanosheets, making them potentially useful in solar energy harvesting and conversion (Fig. 1B). Moreover, PDMM acts as a functional photothermal nanoscale additive, while the CNCs serve as a mechanical reinforcement material [38]. The macro–nanocomposites were carefully prepared in a controlled manner using an advanced freeze-casting method [39]. Overall, owing to the oriented crystallization of ice crystals, the ACPMA evaporator exhibits a uniform and complete vertical channel structure, which enhances water transfer and reduces salt deposition [10,28,40,41]. As shown in Fig. 1C, the ACPMA evaporator demonstrates superior performance in photothermal conversion, thermal regulation, water supply, mechanical durability, and hydraulic stability when compared to conventional evaporators, as evidenced by its multidimensional advantages highlighted in the radar chart analysis (Table S1). This advancement underscores the significance of the ACPMA evaporator as a highly efficient and stable evaporator for practical applications.

### Structural performance of the ACPMA evaporator

**Fig. 2.**
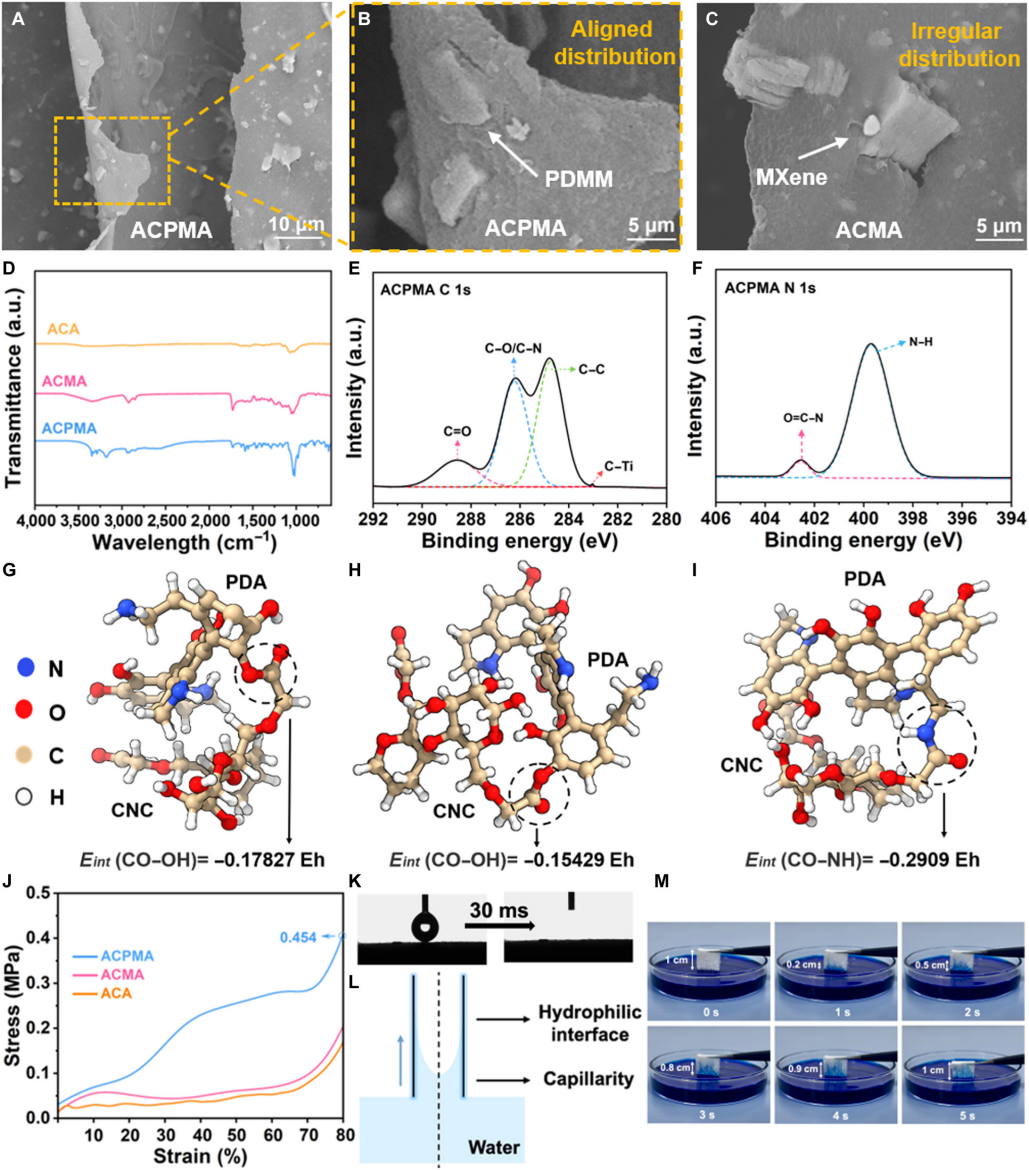
Structure and interfacial interaction characterization of ACPMA. (A) High-magnification scanning electron microscopy (SEM) image of ACPMA’s longitudinal section. (B) High-magnification SEM image of the ACPMA channel wall. (C) High-magnification SEM image of the channel-aligned CNC/MXene aerogel (ACMA) channel wall. (D) Fourier transform infrared (FTIR) spectra of ACPMA, ACMA, and channel-aligned CNC aerogel (ACA). (E) X-ray photoelectron spectroscopy (XPS) chemical analysis of C 1s of ACPMA. (F) XPS chemical analysis of N 1s of ACPMA. (G to I) Binding energy calculations for PDA/CNC. (J) Stress–strain curves of ACPMA, ACMA, and ACA with 80% content. (K) Photographs capturing a single droplet of water before and after. (L) Diagram of capillary force in a vertical channel. (M) Water transportation height of ACPMA as a function of time.

The surface and interior structure of the ACPMA evaporator were revealed via scanning electron microscopy (SEM). The results display that ACPMA has a honeycomb-like architecture with 50- to 300-μm channel dimensions, as revealed by the cross-sectional image (Fig. S1). In Fig. 2A, the layered channel wall structure is flat and dense, as revealed by the longitudinal view, and there are no voids on the surface of the channel walls. Figure 2B presents a magnified view of the channel walls, proving that the PDMM nanosheets are uniformly aligned, effectively dispersed, and securely anchored within the CNC/polyurethane networks. For comparison, a channel-aligned CNC/MXene aerogel (ACMA) and a channel-aligned CNC aerogel (ACA) were also prepared. It can be seen from the electron microscopy image of ACMA that the inner channel wall is incomplete, and there are some voids that are clearly evidenced in Fig. 2C and Fig. S2. This discovery demonstrates that the interfacial force between the MXene nanosheets and the CNCs is too low; the PDA bridging approach can enhance the interfacial force, thereby ensuring both structural integrity and good functional properties [42]. The Fourier transform infrared spectra shown in Fig. 2D reveal a shift in the absorption peak with respect to that of ACMA from 3,347 to 3,323 cm^−1^, which results from additional hydrogen-bonding interactions at the PDA–cellulose interface, as well as between PDA and MXene within the ACPMA system. The peak at 1,632 cm^−1^ corresponds to the C–O stretching in *o*-quinone groups formed via PDA oxidation, while the peak at 1,036 cm^−1^ corresponds to the C–O bond in PDA, and the new peak located at 3,181 cm^−1^ is related to N–H bond stretching. To better explore the effect of PDMM on the CNC-based polymer networks, the surface chemistry of ACPMA was characterized via x-ray photoelectron spectroscopy (XPS). The high-resolution C 1s, O 1s, and N 1s spectra are shown in Fig. 2E and F. The C 1s spectrum shows the presence of C=O (288.5 eV), C–O/C–N (286.3 eV), C–C (284.8 eV), and C–Ti (283 eV), confirming the existence of the PDA layer. In addition, the high-resolution spectrum of O 1s presents 2 deconvoluted spectral features positioned at 533.1 and 532.4 eV, proving the presence of C–O and C=O of ACPMA, respectively (Fig. S3). The high-resolution spectrum of N 1s exhibits 2 deconvoluted peaks, 399.6 eV for N–H and 402.5 eV for O=C–N, respectively. These analyses reveal that PDA can achieve successful cross-linking with CNCs through hydrogen bonding and covalent bonding.

To further elucidate the interaction between PDA and the CNC network, DFT was implemented to determine the reaction energy between CNCs and PDA to confirm the stability of this system [43]. Figure 2I illustrates that PDA forms covalent bonds with CNC networks via catechol/amino groups (–OH/–NH_2_) interacting with CNC carbonyl groups (–C=O), simultaneously promoting solid–liquid interfacial assembly. As shown in Fig. 2G and H, the binding energy of CO–NH (−0.2909 Eh) is stronger than that of CO–OH at various locations (CO–OH_1_: −0.17827 Eh; CO–OH_2_: −0.15429 Eh), suggesting that CNCs react more likely with –NH_2_ than –OH. PDA modification of MXene nanosheets strengthens the interactions with CNCs through covalent cross-linking, which effectively restricts the slip of nanofillers and optimizes stress transfer efficiency. This mechanism imparts better adhesiveness and mechanical stability to MXene nanosheets while simultaneously endowing ACPMA with a well-defined morphology and stable mechanical properties [16,19,33,37]. Owing to the PDA-bridged nanostructure and anisotropic vertical channel structure, ACPMA exhibits excellent compression strength (0.454 MPa) under 80% strain, significantly surpassing that of ACMA in Fig. 2J. As illustrated in Fig. S4, during 30 compression cycles at 80% strain, compression stress decreased during the initial cycle but rapidly stabilized in subsequent cycles, maintaining this remarkably stable state throughout all 30 cycles. This stability indicates that ACPMA has exceptional resistance to compressive fatigue.

To better demonstrate the successful construction of the vertical channel structure, the ACPMA evaporator was investigated via x-ray computed tomography imaging, which reveals a porous structure (Fig. S5). The complete ice crystal growth process is shown in Video S1. Furthermore, the highly porous microstructure of the ACPMA evaporator demonstrates outstanding lightweight properties, with a porosity reaching 87.3% and an ultralow density of 0.0988 g/cm^3^ (Figs. S6 to S8). Overall, the unique vertical channel structure synergistically enhances water transport by combining high porosity, large pore sizes, and a broad pore size distribution. This integrated design enables rapid water transportation and efficient ion diffusion, thereby effectively suppressing salt crystal deposition [38,44,45]. Water contact angle measurements (Fig. 2K) demonstrated the ACPMA evaporator’s superhydrophilic property, with complete droplet absorption occurring in 30 ms (*θ* = 0°). The capillarity and strong hydrophilicity of the vertical channels ensure rapid water transportation (Fig. 2L). Subsequently, an experiment was conducted to evaluate the water transport capabilities of ACA. Specifically, ACA was immersed in methylene blue (MB) solution, and the progression of the water flow was meticulously studied through the digital images presented in Fig. 2M. Notably, the MB solution is pumped rapidly from the bottom, reaching the top within 5 s, thus confirming the remarkable water transport capacity of the vertical channel structure.

### Photothermal performance and mechanism of the PDMM nanosheets

**Fig. 3.**
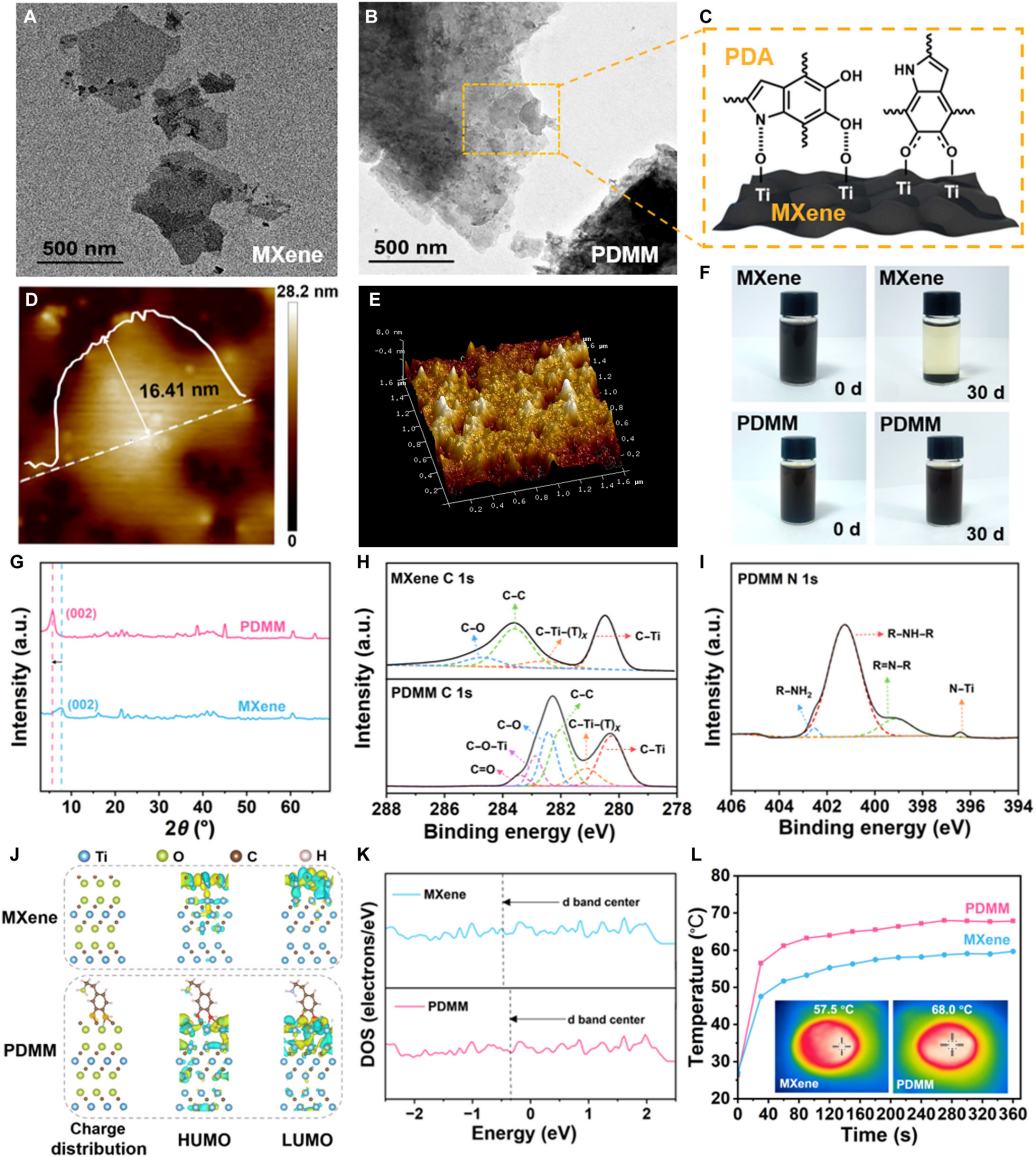
Photothermal performance and mechanism of the PDMM nanosheets. (A) Transmission electron microscopy (TEM) image of MXene nanosheets. (B) TEM image of PDMM nanosheets. (C) Binding mechanism diagram of PDMM. (D) Atomic force microscopy (AFM) image of PDMM nanosheets. (E) Three-dimensional (3D) topography AFM image of PDMM nanosheets. (F) Digital images of MXene and PDMM aqueous dispersions stored at ambient temperature for 30 d. (G) X-ray diffraction (XRD) patterns of MXene and PDMM. (H) XPS chemical analysis of C 1s of PDMM; MXene as control. (I) XPS chemical analysis of N 1s of PDMM. (J) Density functional theory (DFT) calculation for optimized configurations. (K) Density of states (DOS) patterns of MXene and PDMM. (L) Temperature change curves of MXene film and PDMM film under 1-sun irradiation from 0 to 360 s.

The initial synthesis involved aluminum layer extraction from the Ti_3_C_2_T*_x_* MAX phase through LiF/HCl-mediated selective etching to produce Ti_3_C_2_T*_x_* MXene nanosheets. The complete etching and layer separation of MXene nanosheets were validated by transmission electron microscopy, as illustrated in Fig. 3A. Then, dopamine monomers were polymerized in situ on MXene surfaces (Fig. 3C), yielding PDMM nanosheets. Figure 3B exhibits the transmission electron microscopy image of the morphology of the obtained PDMM nanosheets. It can be observed that some thin nanosheets are stacked parallel to each other, which may be attributed to the binding effect of dopamine. Atomic force microscopy provides further evidence of this effect, as shown in Fig. 3D and Fig. S10. After the modification of PDA, the thickness of the nanosheets increased from 8.26 to 16.41 nm, demonstrating that PDA has successfully coated the MXene nanosheets. Additionally, the 3D atomic force microscopy morphology images unveil the height variations and uniform appearance of MXene and PDMM, as shown in Fig. 3E and Fig. S11. After the incorporation of dopamine, PDA wraps the surface of MXene homogeneously. The zeta potential of the bare MXene nanosheets is −13.4 mV, while the zeta potential of the PDMM nanosheets is lower (−43.1 mV) due to the negative PDA charges (Fig. S12). This phenomenon indicates that the surface charge density of PDMM is higher than that of MXene, enhancing the electrostatic repulsion among nanosheets, which suppresses the aggregation of nanoparticles and thus improves stability. By dispersing equivalent quantities of MXene and PDMM in an aqueous medium (Fig. 3F), it can be seen that the MXene solution exhibits discoloration and stratification at room temperature after 30 d, whereas the PDMM solution exhibits no significant changes. These observations further confirm that PDA can inhibit the oxidation of MXene, thereby highlighting the environmental stability of PDMM nanosheets.

The PDA modification mechanism of MXene nanosheets was confirmed via XPS and x-ray diffraction. In Fig. 3G, the strong peak corresponding to the (002) plane is located at 7.52°, verifying the controllable synthesis of MXene nanosheets. The (002) peak of the PDMM nanosheets is shifted to 5.78°, indicating expanded interlayer distances caused by the modification of PDA. Furthermore, XPS spectra display the presence of the C, N, O, Ti, and F elements in PDMM (Fig. S13). The high-resolution N 1s spectrum of PDMM shown in Fig. 3I indicates the bonding state of nitrogen after PDA modification. Therein, the peak at 401.2 eV is attributed to secondary amines (R–NH–R), which dominate the element of N with small amounts of tertiary amines R=N–R (399.2 eV), primary amines R–NH_2_ (402.8 eV), and N–Ti bonds (396.4 eV). Furthermore, as depicted in the high-resolution C 1s spectrum (Fig. 3H), in comparison to MXene, 2 new peaks appear at 282.9 and 283.5 eV. These peaks are attributed to the formation of a catechol–titanium coordination bond (C–O–Ti) and the presence of a quinone state (C=O). These results clearly demonstrate the interaction between amino groups and catechol with the surface of MXene, confirming that dopamine can tightly cross-link MXene sheets. In addition, the 2 new peaks that appear in the PDMM spectrum at 281 and 282.1 eV are assigned to the C–Ti and C–Ti–(T)*_x_* bonds, respectively, and the fact that the binding energies shift toward lower values is likely owing to the electron transfer from PDA to MXene. Additionally, the density of the electron cloud is closely correlated with light absorption and conversion abilities [11].

To reveal the light absorption mechanism and photothermal conversion properties of PDMM, the electronic states of MXene and PDMM were simulated via DFT calculations, as shown in Fig. 3J. The theoretical calculation results reveal that PDMM possesses a significantly higher energy of the highest occupied molecular orbital (3.7494 eV) compared with that of MXene (2.7918 eV). This suggests that electron transfer is easier in PDMM. Additionally, PDMM exhibits a much higher energy of the lowest unoccupied molecular orbital (4.1382 eV), indicating that it readily absorbs electrons and thereby enhances its conductivity. Furthermore, Fig. 3K presents the density of states profiles for MXene and PDMM. In comparison to MXene, the d band of PDMM is shifted toward a higher-energy region, leading to enhanced electron conductivity and improved light absorption efficiency within these specific wavelength ranges. Therefore, with the aim of evaluating the photothermal conversion performance, equivalent quantities of MXene and PDMM were filtered into films on filter paper, and the temperature variations under 1-sun irradiation (1 kW m^−2^) within the NIR spectrum (Fig. 3L) were then examined every 30 s by utilizing an infrared camera. After 6 min of irradiation, the temperature of PDMM rose to 68 °C, while that of MXene was 57.5 °C. This result confirms the superior photothermal conversion properties of PDMM, which mainly arise from the cooperative photothermal effects of PDA and MXene. Specifically, the black rough surface of PDMM improves its solar energy absorbance, prompting PDA to release heat through thermal vibrations, while MXene induces an LSPR effect to release thermal energy, transferring the released heat to PDA and the surrounding MXene. Both simulation and experimental results confirm that the combined photothermal conversion mechanisms greatly enhance solar energy absorption, and thus, more heat can be converted.

### Thermal management and evaporation performance of the ACPMA evaporator

**Fig. 4.**
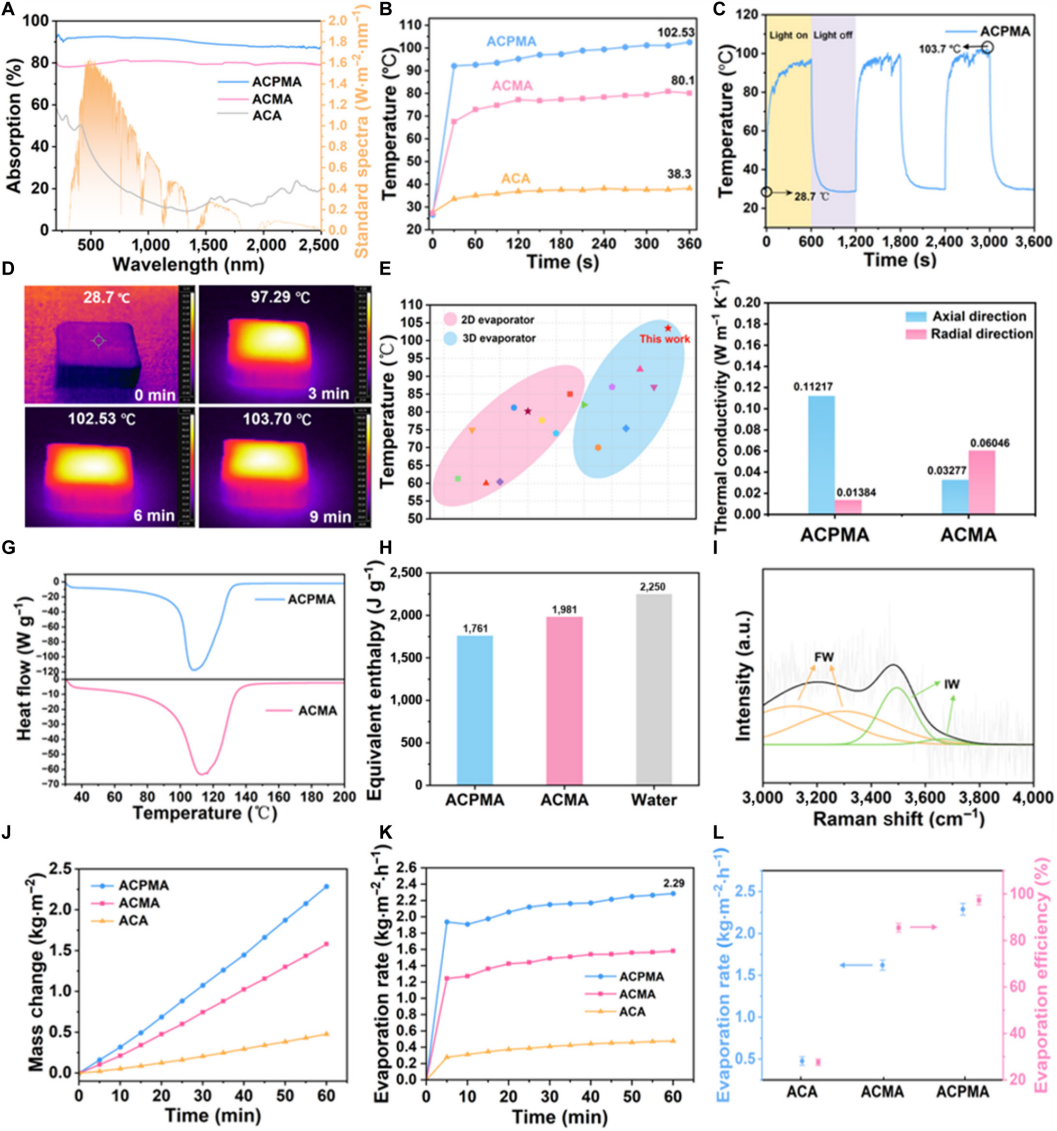
Thermal management and evaporation performance of ACPMA. (A) ultraviolet–visible–near-infrared (UV–Vis–NIR) spectra across 400 to 2,500 nm (AM 1.5 G). (B) Thermal response curves under 1-sun irradiation from 0 to 360 s. (C) Temperature change curves of ACPMA in cycle test under 1-sun irradiation. (D) Infrared image of the rapid heating process of ACPMA under 1-sun irradiation. (E) Comparison of the photothermal performance of this work with those of previously reported evaporators. (F) Anisotropic thermal conductivity of the ACPMA evaporator. (G) Differential scanning calorimetry (DSC) thermograms of water in ACPMA and ACMA (heat-flow-proportional signal). (H) The equivalent enthalpy of water in ACPMA, ACMA, and pure water is obtained from the DSC measurement. (I) Raman spectra showing the fitting peaks of different water states in the ACPMA evaporator. (J) Mass change of simulated seawater with the ACPMA, ACMA, and ACA evaporators under 1-sun irradiation. (K and L) Evaporation rate and efficiency of the ACPMA, ACMA, and ACA evaporators. FW, free water; IW, intermediate water.

Benefiting from the enhanced photothermal performance of PDMM and the exceptional light-capturing capability of the vertical microstructure, the ACPMA evaporator possesses broadband solar absorption and superior photothermal conversion performance. The light absorption of ACPMA, ACMA, and ACA in the range from 400 to 2,500 nm was measured via ultraviolet–Vis-NIR spectroscopy (Fig. 4A). Compared with ACMA, the light absorption of ACPMA is improved after PDA modification, with a remarkable light absorption percentage of ~94.7% across the whole spectrum. This result primarily stems from the energy excitation of the PDA molecules for a rapid π–π* transition [18,19] and the high light-trapping capability of the 3D vertically aligned pore structure [46–48]. The strong light absorption capability forms the basis for achieving a highly efficient photothermal conversion. Under 1-sun irradiation in air, ACPMA demonstrates exceptional photothermal response with 60-s heating from 26.7 to 92.6 °C. The increase in PDMM nanosheets of ACPMA improves its photothermal conversion efficiency compared with that of ACMA, and ACPMA can reach 102.53 °C within 360 s (Fig. 4B). Moreover, the photothermal response significantly contributes to the effectiveness of evaporators. As shown by the infrared image in Fig. 4D, the surface temperature of ACPMA attains a peak of 103.7 °C after 9 min and remains stable at approximately 103 °C under 1-sun illumination. Upon switching off the solar simulator, the temperature rapidly decreases and returns to ambient temperature within 10 min (Fig. 4C). Upon repeating these heating/cooling cycles 3 times, the evaporator remains light sensitive, indicating its stable and superior photothermal conversion performance compared with that of other evaporators (Fig. 4E), laying the foundation for the fabrication of efficient and stable evaporators [15,20,21,49–58]. The thermal conductivity in solar steam generators is also essential for maximizing the useful energy output. In the axial direction, ACPMA exhibited a thermal conduction value of ~0.11 W m^−1^ K^−1^ in dry state, higher than that of ACMA (~0.03 W m^−1^ K^−1^). However, along the radial orientation, the thermal conductivity of ACPMA was 0.01 W m^−1^ K^−1^, which is lower than those of ACMA (~0.06 W m^−1^ K^−1^), pure water (~0.60 W m^−1^ K^−1^), and MXene (~0.35 W m^−1^ K^−1^), as shown in Fig. 4F. These results indicate that the superior axial thermal conductivity between water and the evaporator surface can significantly enhance evaporation efficiency. Additionally, the reduced radial thermal conduction indicates that the hierarchical structure minimizes interfacial thermal losses and restricts conductive losses to the surrounding bulk water [59]. Therefore, it is confirmed that the evaporator exhibits exceptional photothermal management performance.

For efficient thermal management, the equivalent enthalpy of evaporation proves equally crucial. The evaporation enthalpies of ACPMA, ACMA, and water were characterized by differential scanning calorimetry (5 °C min^−1^, N_2_ atmosphere) to assess water molecular states. The differential scanning calorimetry results indicate that the endothermic peak area of ACPMA is smaller than that of ACMA, suggesting that ACMA requires more thermal energy to produce the same mass of steam during evaporation, as shown in Fig. 4G and Fig. S14. Furthermore, by integrating the endothermic peak areas of the 2 samples, the evaporation enthalpy of the water inside ACMA was 1,981 J g^−1^, much higher than that of the water inside ACPMA (1,761 J g^−1^), revealing the significantly lowered vaporization enthalpy of water compared with that of bulk water (Fig. 4H). To better elucidate this phenomenon, different water states in ACPMA and ACMA were analyzed by Raman spectroscopy (Fig. 4I) [60]. The water confined within the evaporator can be classified into bound water, intermediate water (IW), and free water (FW). The Raman spectrum of the water in the ACPMA evaporator reveals 2 FW peaks at ~3,131 and ~3,302 cm^−1^, exhibiting quadruple hydrogen-bonding interactions (dual proton-donation and electron-pair acceptance). By comparison, the IW peaks located at ~3,493 and ~3,667 cm^−1^ are weak hydrogen bonds connecting the surrounding water molecules [61]. Therefore, IW is significantly “activated” and requires less energy compared with FW, thus proving the immense potential in terms of the evaporation performance and energy utilization efficiency of the ACPMA evaporator.

During the desalination process (Fig. S15), the mass changes of ACPMA, ACMA, and ACA in simulated seawater (3.5 wt.%) were monitored under 1-sun irradiation (Fig. 4J). Evaporation rates were obtained from the time-dependent mass change gradients (Fig. 4K). ACPMA shows the highest solar evaporation rate (2.29 kg m^−2^ h^−1^), which is higher than those of ACMA (1.66 kg m^−2^ h^−1^) and ACA (0.47 kg m^−2^ h^−1^). To quantitatively compare the various evaporation performances, the corresponding solar–thermal conversion efficiency was assessed using the equation presented in the Supplementary Materials. ACPMA’s evaporation efficiency was calculated to be 97.34% (Fig. 4L), which exhibits an enhancement compared to those of ACMA (85.45%) and ACA (27.65%). To investigate the performance of the evaporator under extreme operating conditions, the ACPMA evaporator was systematically evaluated for its evaporation characteristics by regulating the solar irradiation intensity. The experimental results demonstrate that under irradiance conditions of 2, 3, and 4 suns, the measured evaporation rates reached 5.17, 7.3, and 9.5 kg m^−2^ h^−1^, respectively (Figs. S16 and S17). The evaporation efficiency shows a near-linear dependence on irradiance intensity, which can be attributed to the improved photothermal conversion efficiency and enhanced water-molecule activation. These findings highlight the promising potential of high-intensity solar applications for advanced evaporation systems. Furthermore, evaporation performance tests of the ACPMA were conducted at various salt concentrations (7, 15, and 20 wt.%), resulting in rates of 2.22, 2.05, 1.92 kg m^−2^ h^−1^, respectively (Figs. S18 and S19). The results indicate that as salinity increases, the evaporation rate of the ACPMA evaporator remains relatively stable, with only a slight decline observed at the 20 wt.% simulated seawater. The ACPMA evaporator, with its exceptional photothermal management and high evaporation efficiency, is a potential candidate for application under extreme operating conditions [27].

### Salt-resistant properties and stable performance of the ACPMA evaporator

**Fig. 5.**
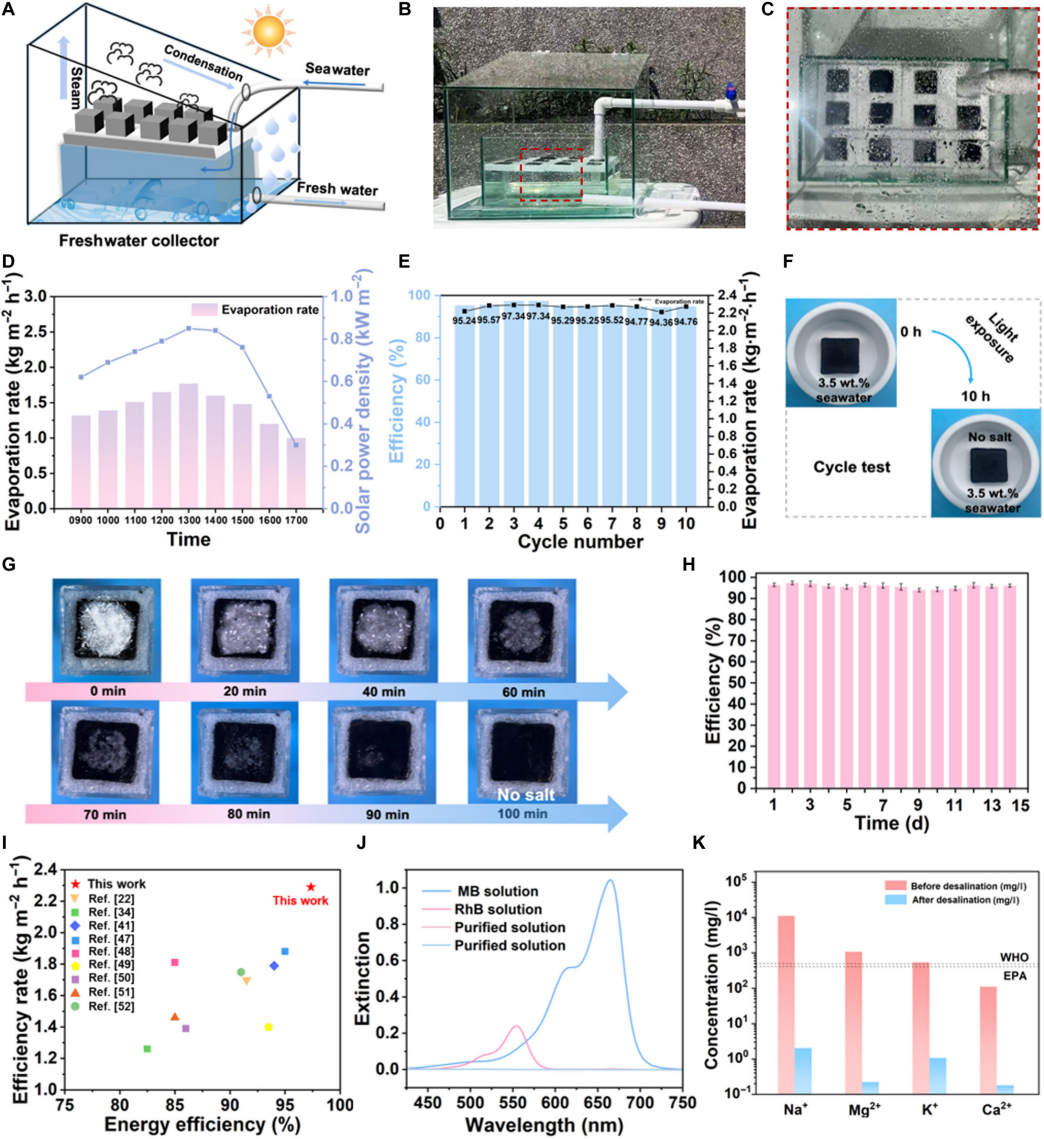
Salt-resistant and stable performance of the ACPMA evaporator. (A) System diagram of the ACPCM freshwater collection device. (B) Digital photo of the freshwater collection device. (C) Digital photo of the top of the freshwater collection device. (D) Evaporation rate under outdoor conditions. (E) Cyclic stability assessment of ACPMA (1 h/cycle) under continuous evaporation conditions. (F) Digital images illustrating the salt-resistant performance of ACPMA evaporators in simulated seawater before and after 10 h. (G) Digital photos showing the salt-resistant performance of ACPMA evaporators after 100 min. (H) Evaporation efficiency of ACPMA over 14 d in simulated seawater under 1-sun irradiation. (I) Comparison of the evaporation performance of this work with those of previously reported evaporators. (J) UV–Vis spectral changes of dye-contaminated water before and after desalination. (K) Ion concentration variations in high-salinity effluent before and after solar desalination. MB, methylene blue; RhB, rhodamine B; WHO, World Health Organization; EPA, US Environmental Protection Agency.

The remarkable evaporation performance of ACPMA makes it an ideal candidate for practical applications in seawater desalination. To evaluate the practical applicability of ACPMA, an outdoor test was carried out for the evaporation of 3.5% salinity simulated seawater in Harbin (126°62′47″E, 45°76′63″N) on 2024 October 1. As shown in Fig. 5A, the ACPMA evaporators were placed in a closed water collection device outdoors. As the seawater undergoes evaporation, steam condenses at the top of the evaporation and water collection device, resulting in the formation of numerous pure droplets, as shown in Fig. 5B and C. The experiment lasted from 0900 to 1700, and the evaporation rate exhibited a positive correlation with solar intensity, reaching the maximum (1.77 kg m^−2^ h^−1^) at 1300 (Fig. 5D). The outstanding evaporator evaporation performance evidenced by the above outdoor test shows the suitability of this evaporator for practical applications.

Notably, long-term stability is a crucial and indispensable factor in practical applications. The exceptional mass transport performance of ACPMA inhibits the accumulation of salt, due to its distinctive vertical channel structure. To demonstrate the excellent stability of the ACPMA evaporator over prolonged operation periods, the evaporator was tested over 10 cycles in simulated seawater, with each cycle persisting for 10 h. The evaporation efficiency consistently remained between 94.36% and 97.34% throughout all cycles (Fig. 5E) [62]. No salt crystals appear on the surface, indicating the salt-resistant properties of ACPMA (Fig. 5F). This phenomenon arises due to the concentration-driven backflow of dissolved salt ions from the saturated solution to bulk seawater through the transport channel [47,48]. During continuous evaporation, salt ions migrate toward the liquid–vapor interface through convective capillary flow within oriented channels, resulting in progressive solute enrichment at the interfacial region. Concurrently, the salinity gradient creates a chemical potential imbalance across the water–evaporator interface, driving the reverse diffusion of ions back into the bulk water. This counteracting diffusion mitigates interfacial solute accumulation, thereby dynamically maintaining the salt concentration in a subsaturated state within the channels during the evaporation process. The balance between these processes establishes a self-regulating mechanism, enabling sustained salt-rejecting performance without external intervention [49]. To better assess the salt-resistant capability of ACPMA, a sufficient quantity of salt was placed on its surface at a concentration of 3.5 wt.% simulated seawater. The amount of salt on the surface gradually decreases and completely disappears after 100 min (Fig. 5G). Furthermore, after a continuous 14-d simulated seawater evaporation test, shown in Fig. S20, ACPMA retains an evaporation efficiency of 93%, and its morphology does not change significantly, which indicates its prolonged stability (Fig. 5H). It is worth noting that the ACPMA system outperforms most reported 3D evaporators in both evaporation rate and efficiency (Fig. 5I) [13,26,31,49,55,62–65]. Moreover, rhodamine B and MB aqueous solutions were also employed in solar evaporation to further evaluate the purification ability of the ACPMA evaporator. As shown in Fig. 5J, spectral analysis clearly identifies characteristic absorption peaks at 554 nm (rhodamine B) and 664 nm (MB). These 2 peaks almost disappear after evaporation, demonstrating the evaporator’s superior water purification performance. Collectively, the complete quenching of dye-specific absorbance peaks and observed solution decolorization (Fig. S21) verify exceptional contaminant removal performance under solar irradiation. Furthermore, the ion concentration of the collected fresh water was carefully quantified via inductively coupled plasma atomic emission spectroscopy (Fig. 5K). The results suggest that the 4 dominating salt ions (namely, K^+^, Ca^2+^, Na^+^, and Mg^2+^) taken from collected seawater after desalination fully conform to the clean water standards set by the World Health Organization and US Environmental Protection Agency. Therefore, these outdoor tests and cycling tests on the salt-resistance properties and stability of the evaporator demonstrate its immense potential in practical applications.

## Discussion

In conclusion, an anisotropic aerogel solar-driven evaporator with simultaneously enhanced photothermal performance and long-term stability was here developed using the MXene–PDA–CNC bridging approach. The integration of PDA not only boosts the photothermal performance of MXene but also increases the interfacial force when cross-linked with CNC, ultimately improving the mechanical properties. Additionally, the wood-like vertical channel structure endows the ACPMA aerogels with exceptional light absorption properties, high mechanical strength, and superior water transport efficiency. Owing to these functional attributes, the anisotropic evaporator exhibits a high evaporation rate of 2.29 kg m^−2^ h^−1^ (1-sun illumination), which is superior to those of traditional solar evaporators. Furthermore, the vertically ordered channel structure promotes both convective transport and ionic diffusion, thus ensuring that the ACPMA evaporator exhibits stable and consistent salt rejection performance over a 14-d period during seawater desalination. This anisotropic ultra-efficient evaporator with long-term stability advances practical solar desalination deployment while innovating the design of high-productivity evaporators.

## Materials and Methods

### Materials

The CNCs were purchased from Guangxi Qihong Technology Co., Ltd. (China). Waterborne polyurethane (WPU) was purchased from Jining Huakai Resin Co., Ltd. (China). Ti_3_AlC_2_ (MAX, 400 mesh) was purchased from Brofos Nano Technology Co., Ltd. (China). Dopamine hydrochloride was purchased from Aladdin Biochemical Technology Co., Ltd. (China). Tris–hydrochloride acid aqueous solution was purchased from Aladdin Biochemical Technology Co., Ltd. (China).

### Preparation of MXene (Ti_3_C_2_T*_x_*) nanosheets

MXene nanosheets were synthesized through a modified hydrofluoric acid etching protocol. Briefly, 2 g of MAX was slowly introduced into 20 ml of hydrofluoric acid (45 °C) under continuous stirring for 56 h. The resulting suspension underwent centrifugation (8,000 rpm, 5 min) followed by repeated deionized water washing until neutral pH (pH ≈ 6) was obtained. The collected supernatant was freeze-dried, yielding organ-like MXene structures.

### Preparation of PDMM

Fifty milligrams of dopamine hydrochloride was dissolved in Tris–hydrochloride acid aqueous solution under stirring (pH ≈ 8.5), and the obtained solution was subjected to a pre-reaction for 1 h. Through controlled dropwise infusion, dopamine hydrochloride (2 mg/ml in aqueous solution) was incorporated into a suspension containing 500 mg of MXene. Under maintained vigorous stirring conditions, the 23-h reaction was completed. Subsequently, the MXene solution modified with dopamine was washed and centrifuged several times to remove the untreated monomers in the solution. The obtained PDA-modified MXene is denoted as PDMM.

### Preparation of the WPU/CNC/PDMM suspension

Twelve milliliters of the CNCs and 4 ml of deionized water were added to 1.32 g of WPU solution. Then, 12 ml of the PDMM suspension was homogenized with the above miscible solution via 12-h stirring. Finally, a well-dispersed stable suspension was obtained.

### Preparation of the ACPMA evaporator

The WPU/CNC/PDMM composite precursor solution was cast into a precooled (−10 °C) polydimethylsiloxane mold mounted on a copper substrate. Subsequently, the sample underwent freezing treatment via liquid nitrogen at a controlled freezing rate (10 °C min^−1^), followed by 72-h freeze-drying and thermal cross-linking at 80 °C for 8 h.

### Preparation of ACMA and ACA

Twelve milliliters ml of CNCs and 4 ml of deionized water were added to 1.32 g of WPU solution. Then, 500 mg of MXene nanosheets was homogenized with the above miscible solution via 12-h stirring. The obtained highly dispersed homogeneous suspension was cast into a precooled (−10 °C) polydimethylsiloxane mold mounted on a copper substrate, with the freezing rate controlled at 10 °C min^−1^ using liquid nitrogen. After 3-d freeze-drying and thermal curing at 80 °C for 8 h, ACA and ACMA were obtained. The preparation of ACA was the same as that of ACMA but without MXene.

## Data Availability

All data supporting the findings of this study are available within the paper and its Supplementary Materials. In addition, the datasets generated or analyzed during this study are available from the corresponding authors on reasonable request.
