# Supplementary material for: Ultra-efficient, Anisotropic Cellulose Aerogel with Polydopamine Interfacial Bridged Structure and Photothermal Modification for Seawater Desalination
Source: Research (Wash D C). 2025 Sep 25;8:0888. doi: 10.34133/research.0888 (PMC12463581; doi:10.34133/research.0888)
Supplement: Supplementary 1 — Figs. S1 to S22 Table S1 Movie S1 [file research.0888.f1.zip › Supporting Information-revised.docx]

Supporting Information

**A. Experimental Section**

**1. Characterization**

The morphologies of nanosheets are observed by transmission tron microscopy (TEM, JEM-2100, Japan) and atomic force microscopy (AFM, Bruker Multimode 8, Germany). The internal microstructure of aerogels is characterized by scanning electron microscope (SEM, Hitachi TM3030, Japan) equipped with energy dispersion spectroscopy (EDS). The specific surface area and porosity are collected by mercury injection tester (MicroActive AutoPore V 9600 2.03.00). Fourier Transform Infrared Spectrometer (FT-IR, Nicolet iS 50, Thermo fisher, USA). The X–ray photoemission spectroscopy (XPS) of sample is characterized by X-ray Photoelectron Spectrometer (XPS, THERMO, USA). The phase composition of the sample was analyzed by X-ray diffractometer (XRD, Bruker D8 ADVANCE, Germany). The stability of the nanoparticles was measured by Dynamic Light Scattering (DLS, Malvern Panalytical, Britain). The state of water molecules in evaporators was characterized by Laser microconfocal Raman spectrometer (Ram, RENISHAW, Britain). The three-dimensional structure of aerogel was analyzed by high resolution 3D X-ray microscopic imaging system (Micro-CT, BRUKER, Germany). The compression test is carried out by universal testing machine (Suns, UTM 2503, China). The porosity of the sample was measured by automatic mercury injection instrument (MIP, Micromeritics AutoPore V 9620, USA). Surface wettability of as-prepared samples is determined by an OCA20 Contact angle measurement system (Data-physics, Germany). A thermal constant analyzer (TPS 2500S, Hot Disk, Sweden) tested sample is conducted to demonstrate the thermal conducting ability of samples. The evaporation enthalpy of ACPMA, ACMA and water were analyzed a Q20 differential scanning calorimetry (DSC) from the USA, conducting measurements across a temperature range from, measured in a nitrogen atmosphere at a heating rate of 5°C min-1 over a temperature range of 20 to 200°C. The UV-visible-NIR spectrophotometer (UV-3600, Shimadzu, Japan) is used to measure the light absorption and the diffuse reflectance UV-Vis spectra of samples. Infrared imaging device (FLIR-E6390, Sweden) is used to track the exterior temperature changes during the light-thermal energy conversion process. A solar simulator (San-EI Electric Co., Ltd, Japan) is used to simulate the solar source in seawater desalination progress. The residual content for main positive ions in the sea water before and after desalination is detected by an inductively coupled plasma source mass spectrometer (ICP-MS, Agilent 7800ce, USA). The ultraviolet and visible spectrophotometer is measured to test the purity of freshwater after desalination (UV-Vis, PerkinElmer, USA).

**1.1. The methodology to determine the** **thermal conductivity of the anisotropic aerogel**

Thermal conductivity was measured using the Test Protocol Hot Disk TPS 2500s instrument for simultaneous measurement of thermal conductivity along multiple directions. The anisotropic aerogel samples were cut into cylindrical shapes (diameter: 25 mm, thickness: 5 mm) to ensure uniform contact with the TPS sensor. Each sample was measured three times transverse thermal conductivity and three times lengthwise thermal conductivity at room temperature (25°C). The average values were reported, with standard deviations provided to assess measurement reproducibility.

**2. Calculation of heat loss during the evaporation process**

**2.1 Radiation loss θ1**

*θ_1_ = Φ/P_in_*

Based on the Stefan-Boltzmann equation: *Φ* = *ε σ (T_1_^4^-T_2_^4^)*, where *ε* is the emittance of the absorbing surface, σ is the Stefan–Boltzmann constant (σ = 5.670373×10^-8^ W m^-2^ K^-4^), T_1_ is the average surface temperature of sola evaporator, and T_2_ is the temperature of surrounding environment. *P_in_* is the solar irradiation intensity. Thus, the radiation loss *θ_1_* of APCM aerogel is about 4%.

**2.2 Conduction loss θ_2_**

*θ_2_ = Q/P_in_*

*Q = C m ΔT*

where *Q* is the heat energy, *C* is the specific heat capacity of water (4.2 J ℃^-1^ g ^-1^), *m* is the weight of water, *ΔT* is the increased temperature of bulk water after 1 hour evaporation. Therefore, *θ_2_* was calculated to 0.58%

**2.3 Convection loss θ_3_**

*θ_3_ = P/P_in_*

Based on the equation of Newton’s law of cooling: *P = h A ΔT*, where *P* denotes the heat energy, *h* represents the convection heat transfer coefficient (5 W m^-2^ K^-1^), *A* is the actual surface area. *ΔT* is the difference between the surrounding environment and the surface temperature of the solar evaporator. Based on the equation, *θ_3_* was calculated to account for 0.45%.

Based on the equations of the three sources of heat loss, the theoretical efficiency of solar vapor evaporation efficiency is about 94.97%, which aligns well with experimental result that is 97.34%.

1. **Calculation of solar evaporation efficiency**

The solar energy conversion efficiency (**η**) can be calculated by the following formula:

$$\eta=\frac{v(H+Q)}{C_{opt}}$$

where ***v*** is solar evaporation rate (2.29 kg m^−2^ h^−1^), ***H*** is evaporation enthalpy and ***Q*** is sensible heat of water (4.2 kJ·kg^-1^·K^-1^), and ***C_opt_*** is the solar irradiation intensity (1 kW m^−2^). Thus, by the equations, the corresponding evaporation efficiency (η=97.34%) under one sun irradiation was obtained.

**4. The oriented channel structure facilitates salt resistance through three synergistic mechanisms:**

**(1) Rapid water transport**

The oriented channel evaporator forms a larger water transfer channel by optimizing the channel diameter (50μm-300μm). Compared with the micro-channel of natural wood (12-50μm), the expansion of its pore diameter significantly increases the water transfer rate. According to the ***Hargen-Poisye*** equation:

$$Q=\frac{\pi\Delta PR^{4}}{8\eta L}$$

The variables in ***Hargen-Poisye*** equation are defined as follows: Q represents the volumetric flow rate (m³/s), ΔP is the pressure difference (Pa) between pipe ends, R denotes the inner radius (m) of the cylindrical pipe, η stands for the dynamic viscosity (Pa·s) of the fluid, and L indicates the length (m) of the pipe.

According to the equation, increasing pore size fundamentally improves the flow rate of water, thereby enabling more efficient convective transport. This enhanced transport promotes the diffusion of salt from the evaporator surface into the bulk water, resulting in more effective salt dilution.

**(2) Enhanced reverse diffusion of salt ions**

The salt diffusion rate is strongly influenced by the porosity. As described by **Fick’s law**, the reflux rate of salt ions scales positively with porosity. Higher porosity enhances diffusion flux, thereby promoting salt dilution and mitigating ion accumulation at the evaporation surface. The porosity of our evaporator (ACPMA) is as high as 87.3%, thus enabling excellent salt diffusion performance.

**(3) Continuous convective outflow maintains sub-saturation conditions.**

Rapid water transport and reverse salt diffusion synergistically establishes a continuous convective transport system, effectively maintaining sub-saturation conditions at the evaporation interface.

**B. Supporting Tables and Figures**

**Table S1.** Comparison between our ACPAM evaporator with other reported evaporators with related factors

| Evaporators | Thermal insulation  (W m^-1^ K^-1^) | Rate  (kg m^-2^ h^-1^) | Efficiency | Strength  (MPa) | Stability | Ref. |
| --- | --- | --- | --- | --- | --- | --- |
| CFM@PDA | / | 1.79 | 92.60% | / | 8 h | [1] |
| MXene/MFs | 0.08987 | 1.54 | 87.10% | / | 10 h | [2] |
| DW-TA-Fe^3+^ | 0.02965 | 1.79 | / | / | 10 h | [3] |
| Bamboo leaves | 0.1 | 1.75 | 91.9% | / | 10 h | [4] |
| Silk fibroin (SF) | / | 2.03 | 94.6% | / | 20 h | [5] |
| KLC/MF  MXene/Au@Cu_2−x_S  MXene/3D honeycomb  CNT-MB@CF  PPy/BiVO_4_-PI/MXene  AgPW | /  /  0.0423  /  /  / | 1.539  2.023  1.62  1.88  1.64  2.04 | 96.5%  96.1%  93.5%  95.47%  96.77%  90.7% | /  /  /  /  0.25  / | 12 h  10 h  35 h  10 days  10 h  8 h | [6]  [7]  [8]  [9]  [10]  [11] |
| SGA  rGO-CuO/Cu_2_O | 0.085  0.07784 | 2.09  2.10 | 85%  83.39% | /  / | 7 days  7 h | [12]  [13] |
| Our work | 0.01384 | 2.29 | 97.34% | 0.454 | 14 days |  |

**
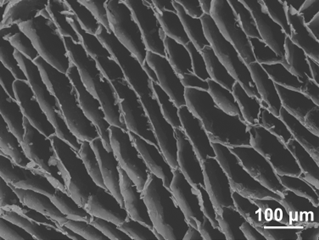
**

**Fig. S1** High magnification SEM image of ACPMA in cross-section view.

**
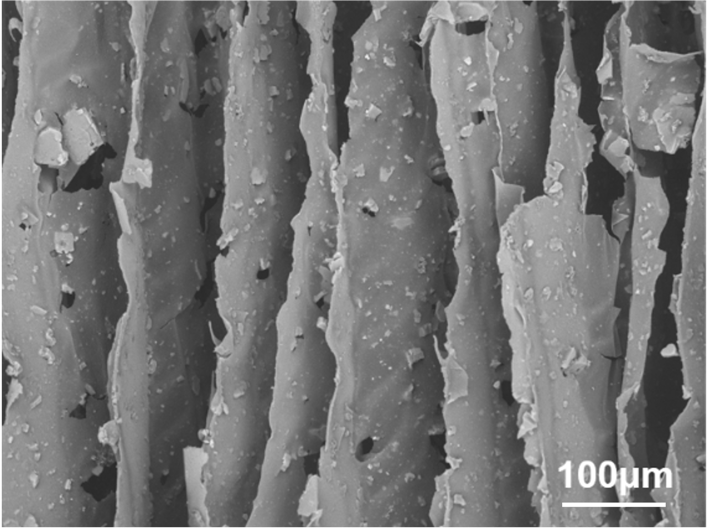
**

**Fig. S2** High magnification SEM image of the ACMA in longitudinal view.

**
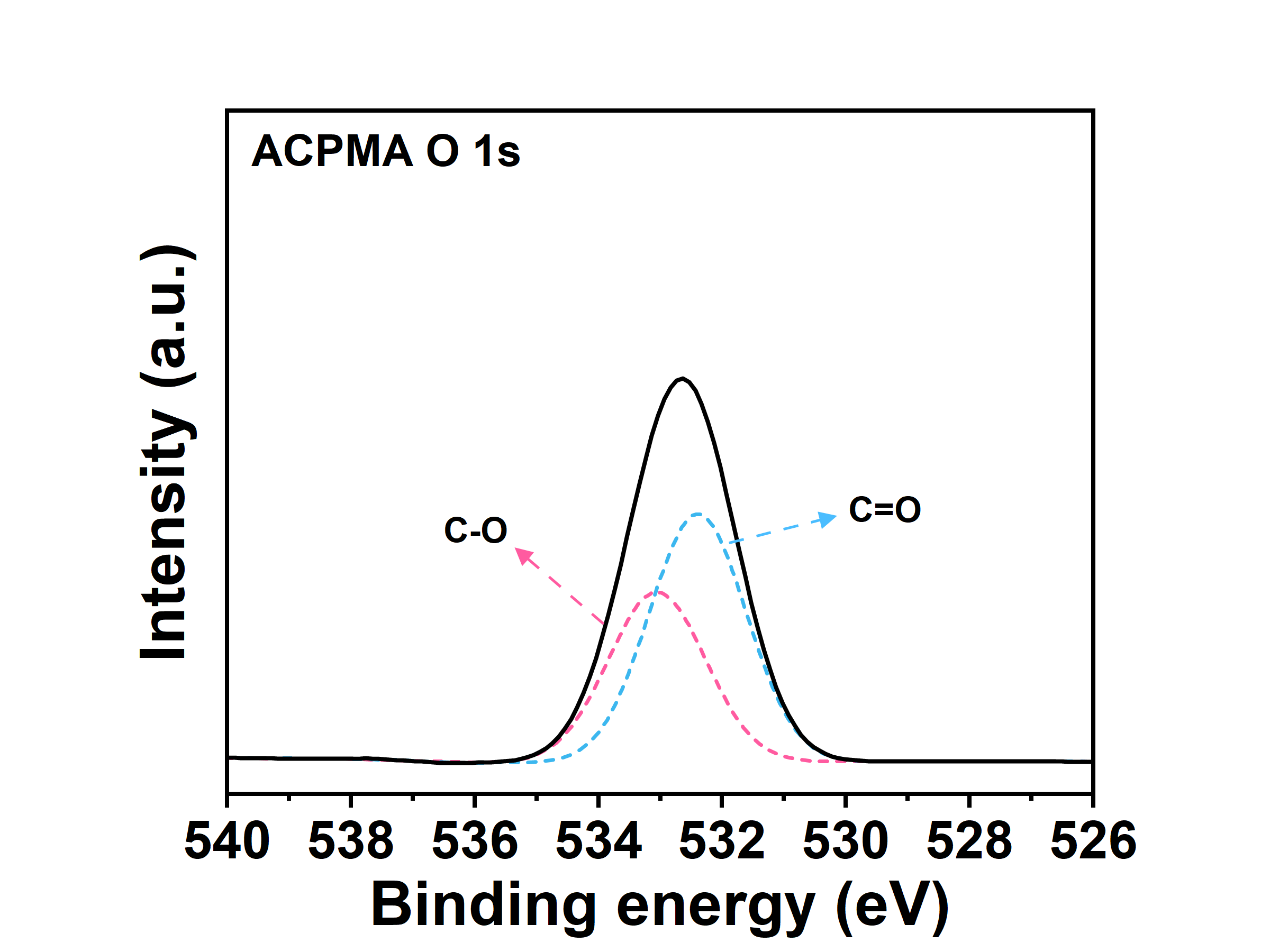
**

**Fig. S3** XPS patterns of O element in ACPMA evaporator.


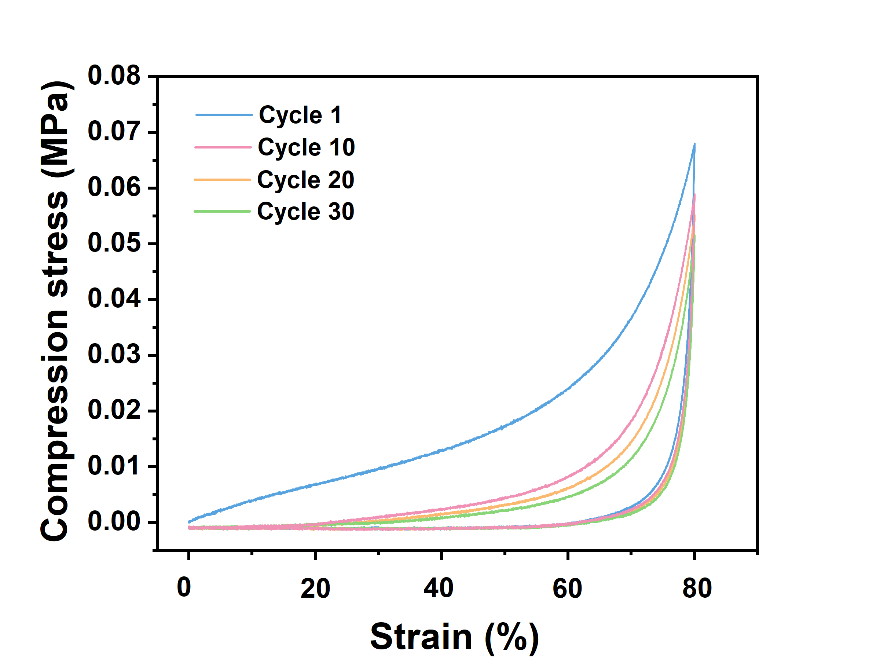


**Fig. S4** Fatigue resistance at 80% strain under compression for 30 cycles.

**
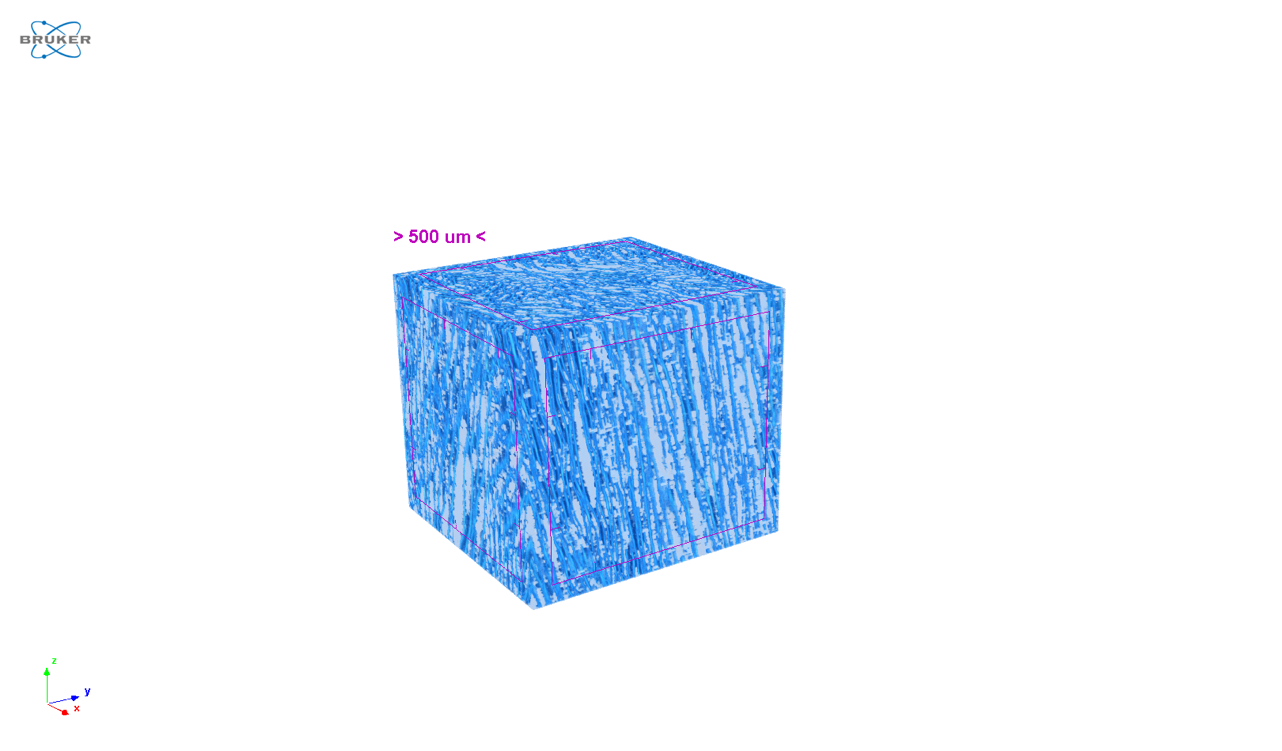
**

**Fig. S5** Vertical channel structure of ACPMA by X-ray computed tomography

**
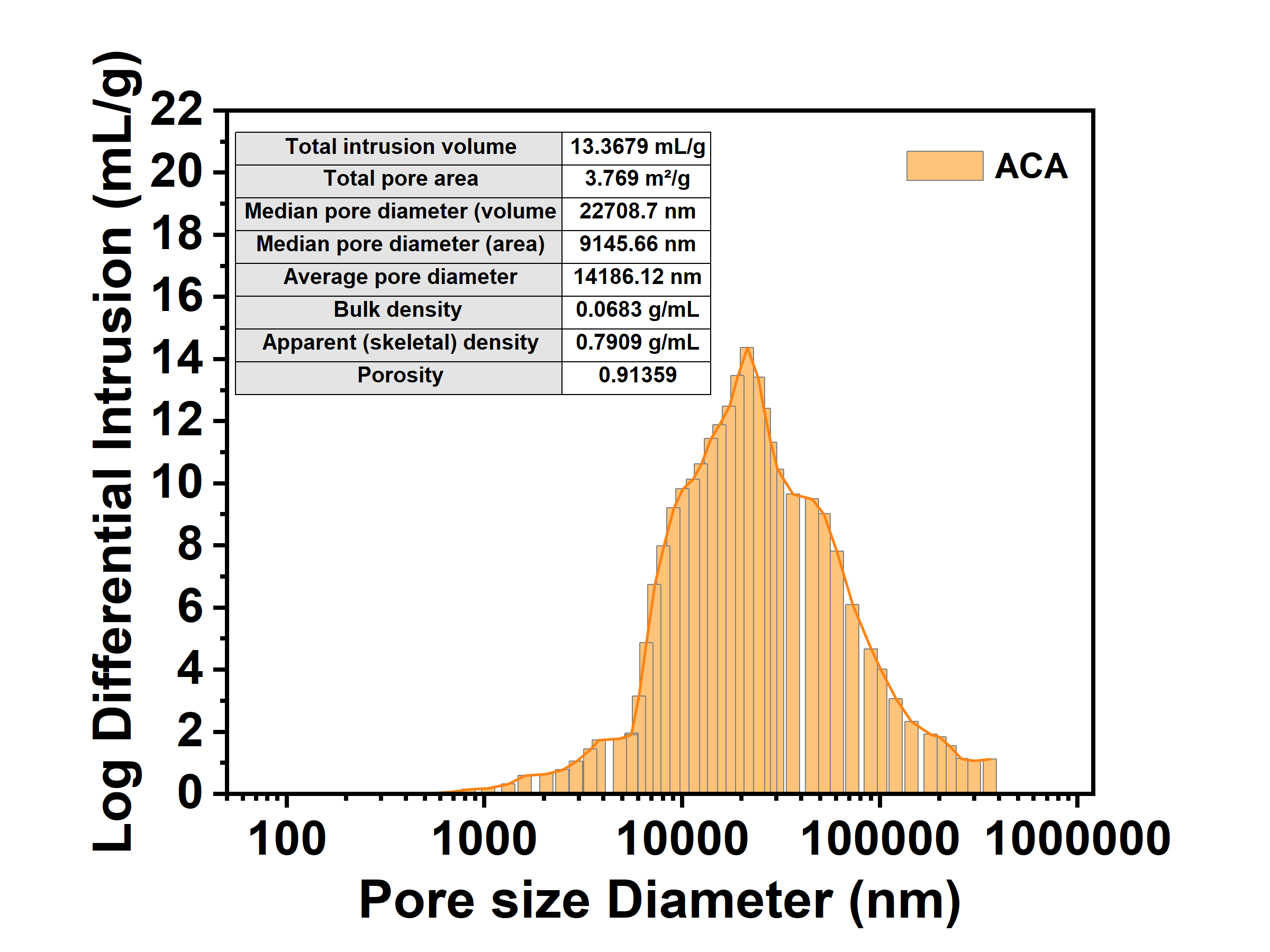
**

**Fig. S6** Pore size distribution and porosity of ACA


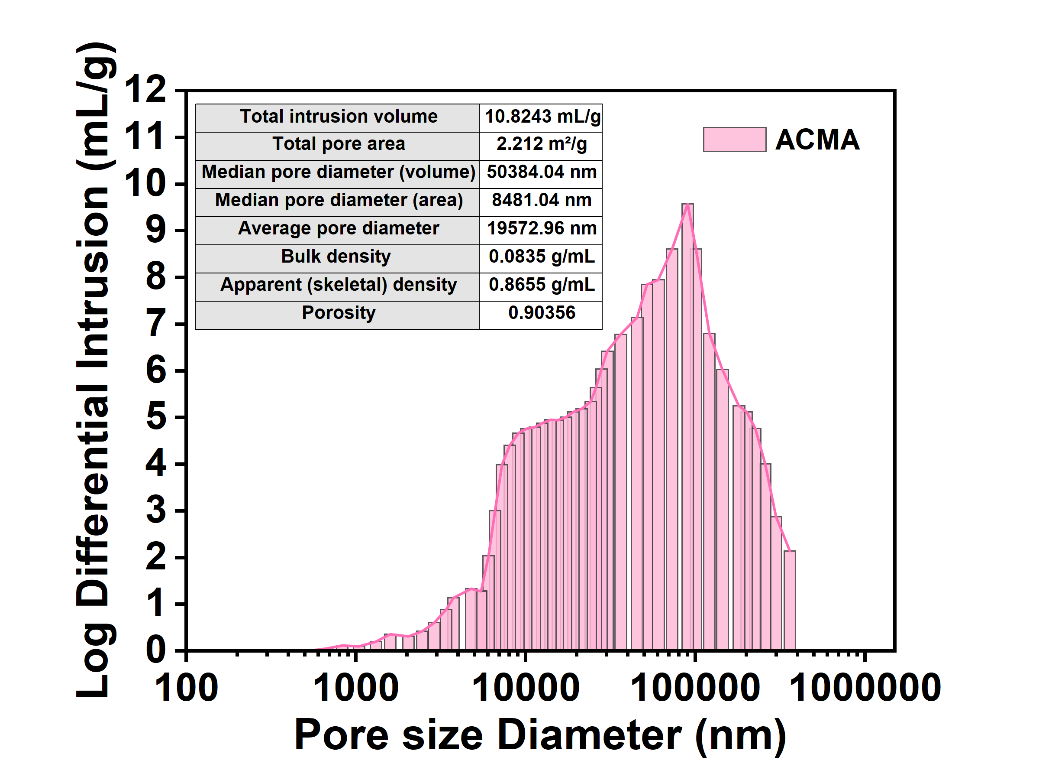


**Fig. S7** Pore size distribution and porosity of ACMA


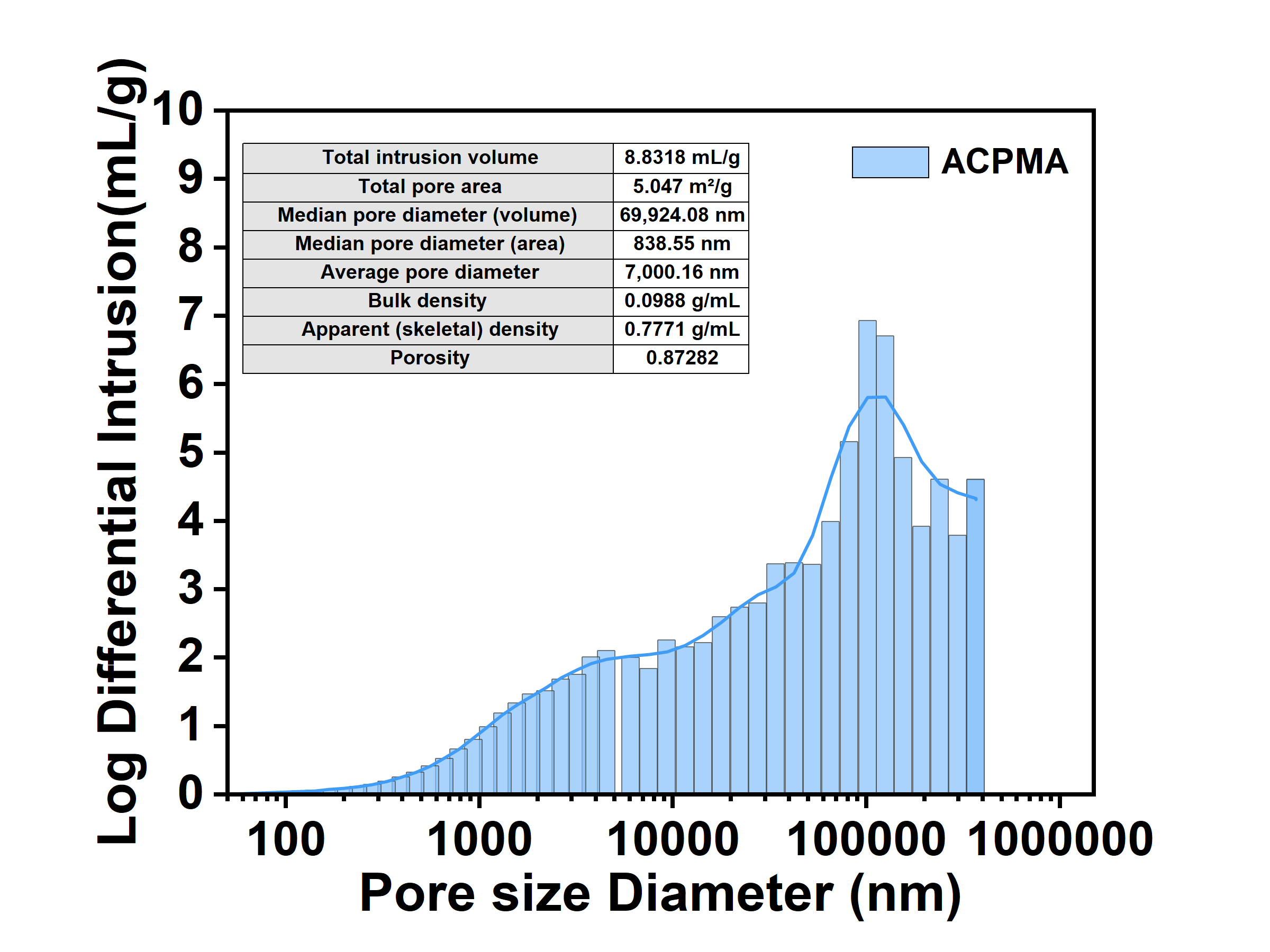


**Fig. S8** Pore size distribution and porosity of ACPMA


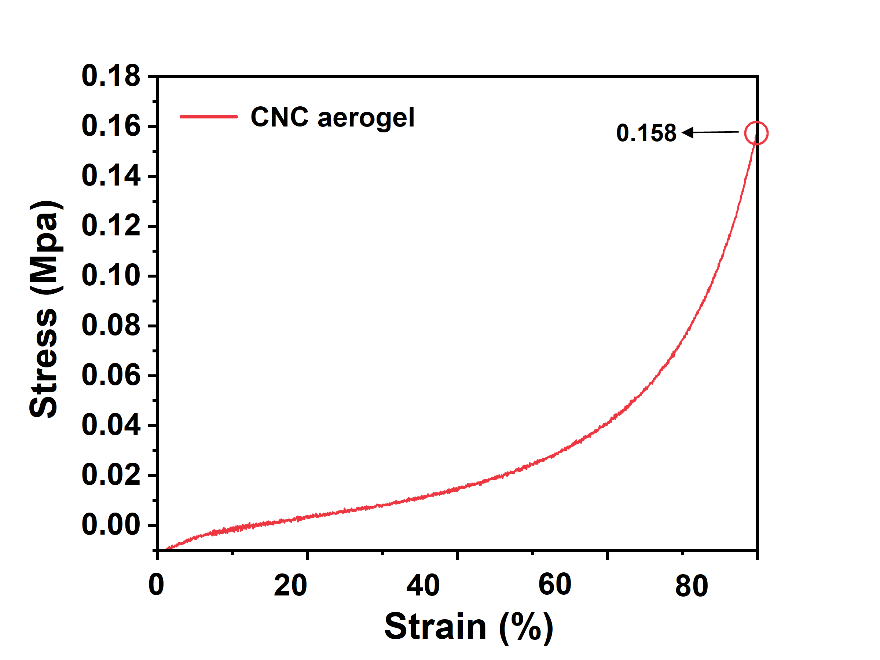


**Fig. S9** Stress–strain curves of CNC aerogel under 80% strain.

**
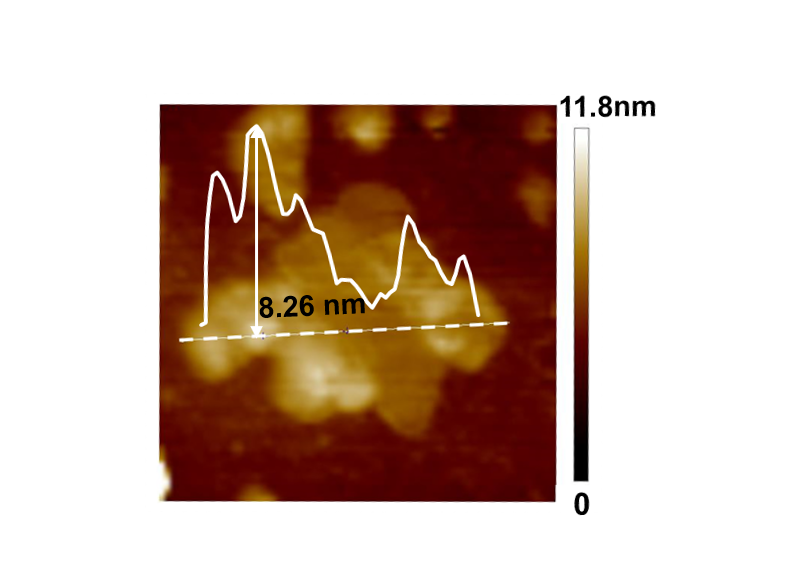
**

**Fig. S10** AFM image of MXene nanosheets

**
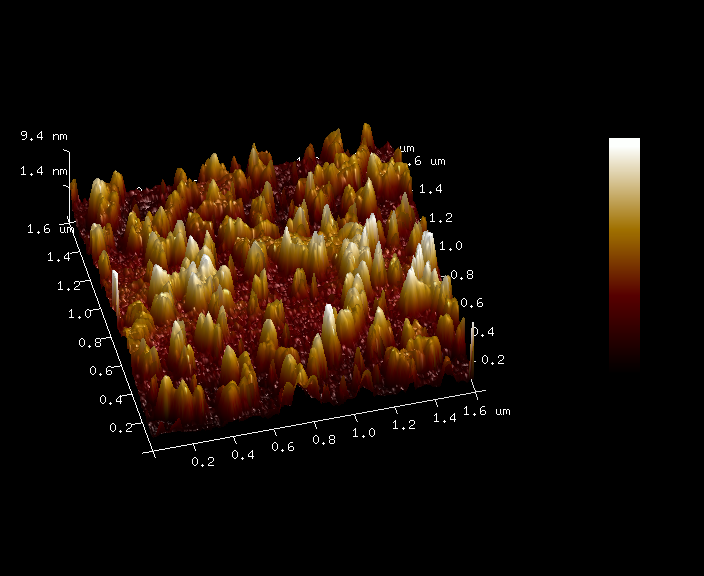
**

**Fig. S11** 3D topography AFM image of MXene nanosheets


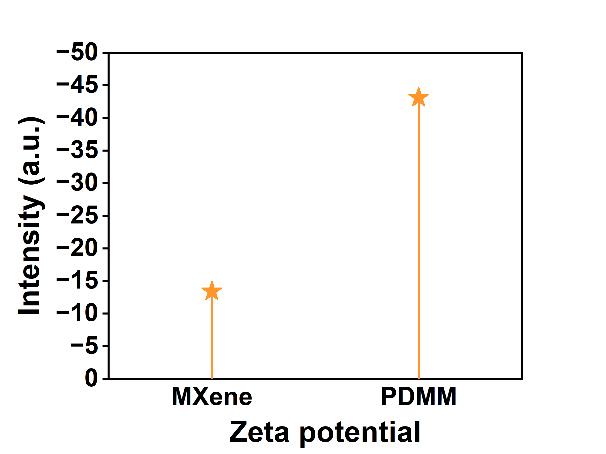


**Fig. S12** Zeta potential diagram of MXene and PDMM nanosheets.

**
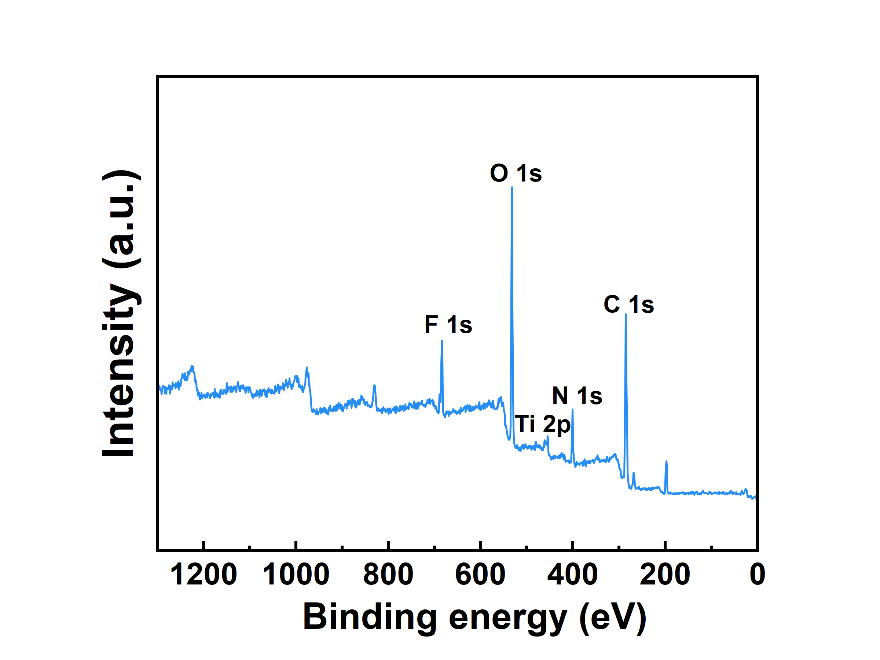
**

**Fig. S13** XPS patterns of C, N, O, Ti, and F elements in PDMM nanosheets

**
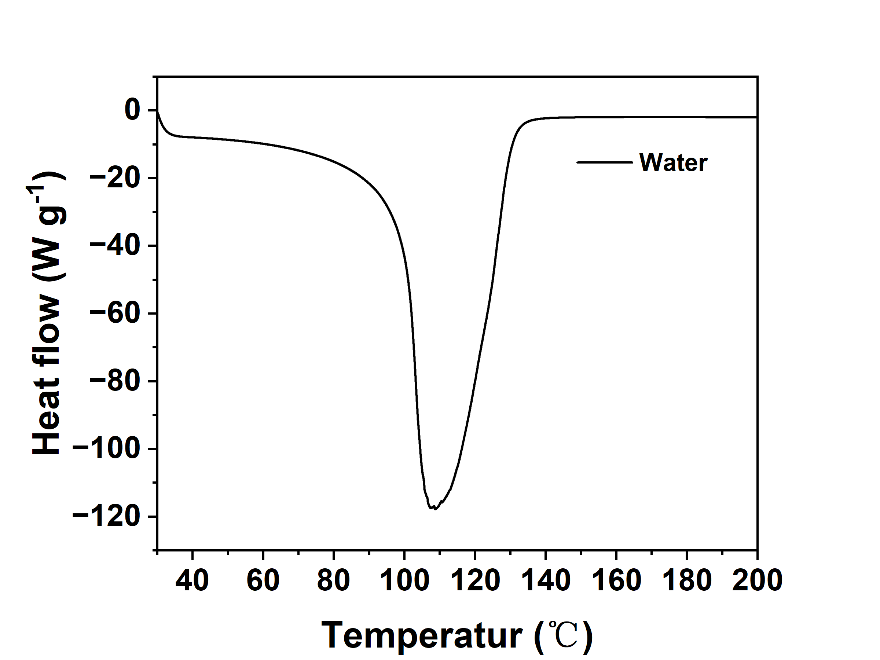
**

**Fig. S14** DSC curve of water

**
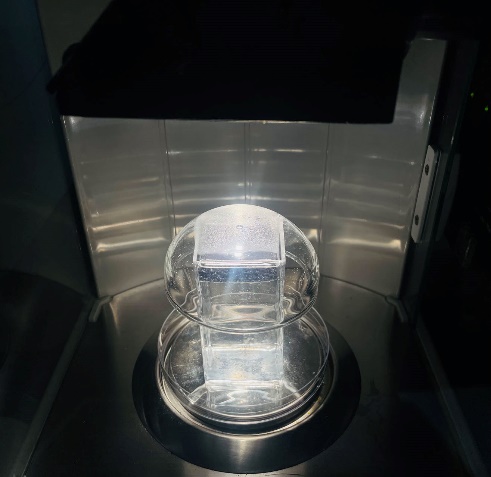
**

**Fig. S15** A diagram of ACPMA in desalination progress under simulated sunlight


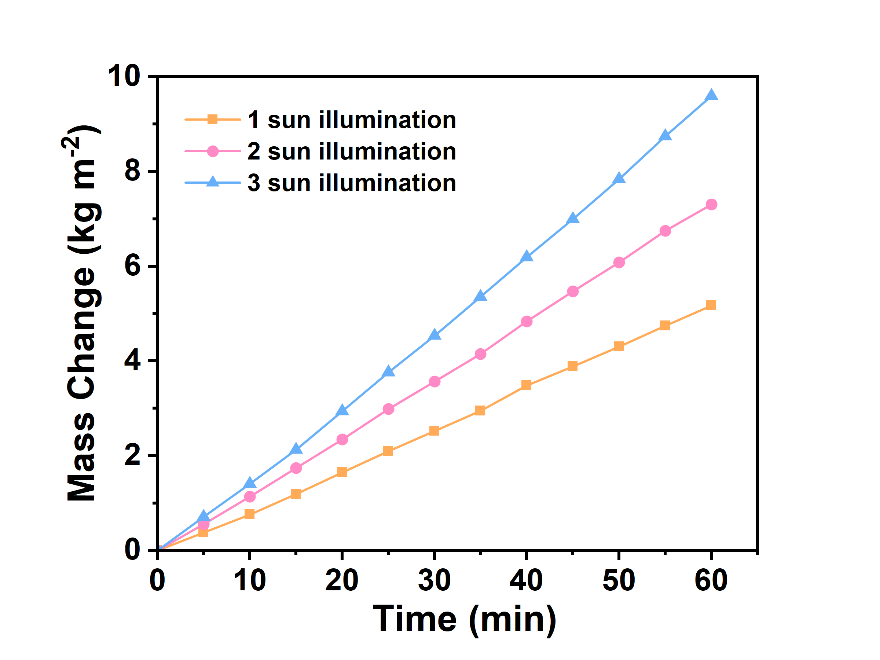


**Fig. S16.** Mass change of simulated seawater in the ACPMA Evaporator with salt concentrations of 7 wt.%, 15 wt.%, and 20 wt.%


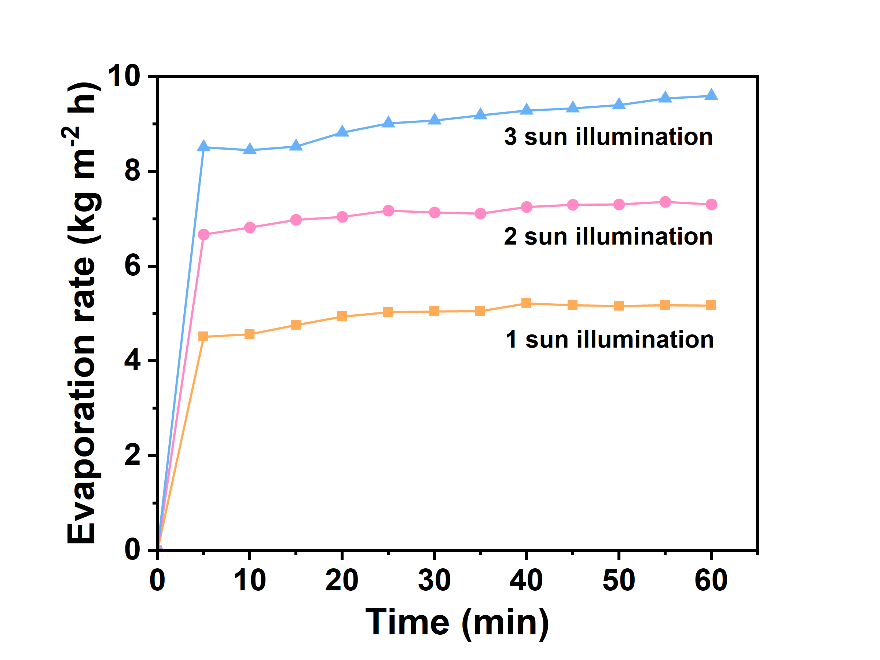


**Fig. S17.** Mass change of simulated seawater in the ACPMA Evaporator with salt concentrations of 7 wt.%, 15 wt.%, and 20 wt.%


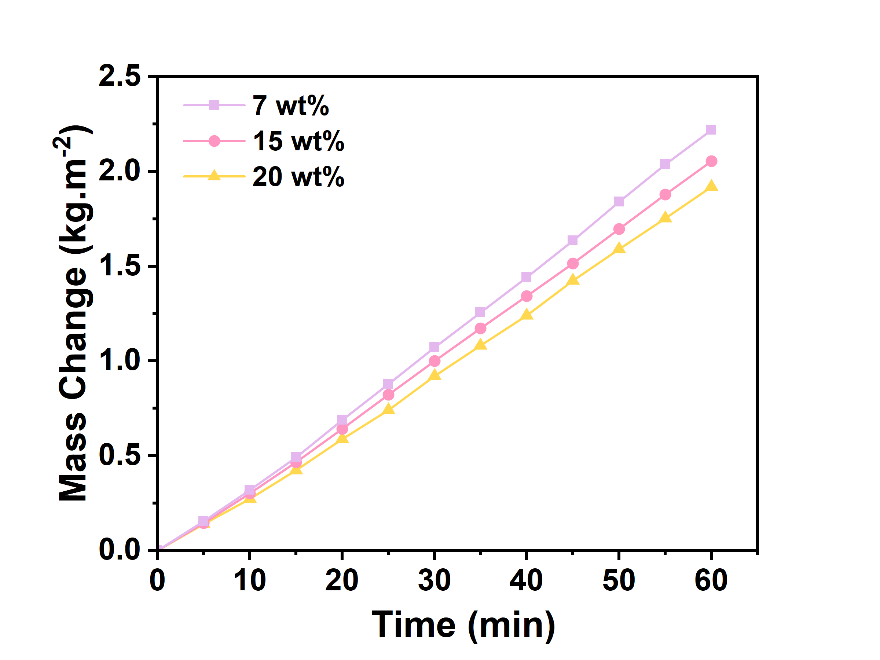


**Fig. S18** Mass change of simulated seawater in the ACPMA Evaporator with salt concentrations of 7 wt.%, 15 wt.%, and 20 wt.%


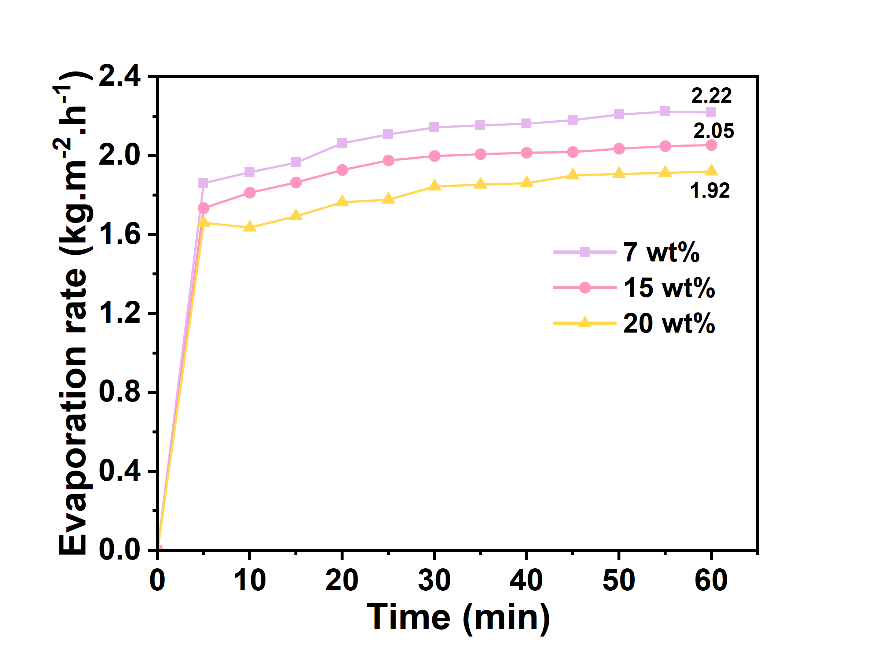


**Fig. S19** Evaporation rate of simulated seawater in the ACPMA Evaporator with salt concentrations of 7 wt.%, 15 wt.%, and 20 wt.%


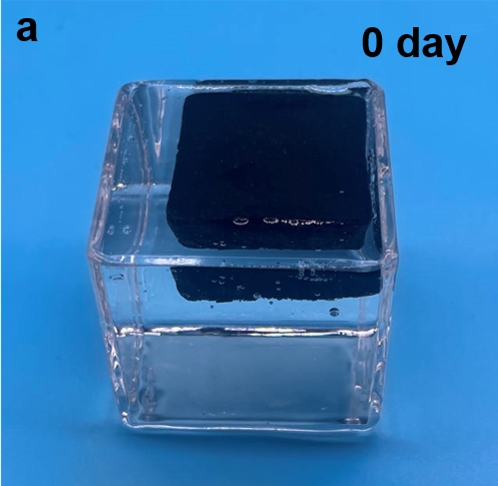

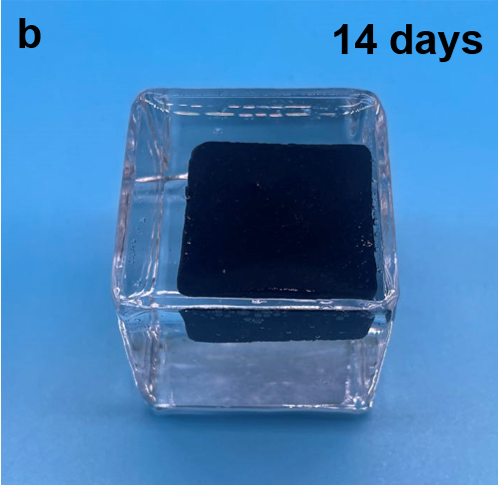


**Fig. S20** Photographs of ACPMA could keep stable in simulated seawater for 14days. **a** Photographs of the state of ACPMA on Day 0. **b** Photographs of the state of ACPMA on Day 14.


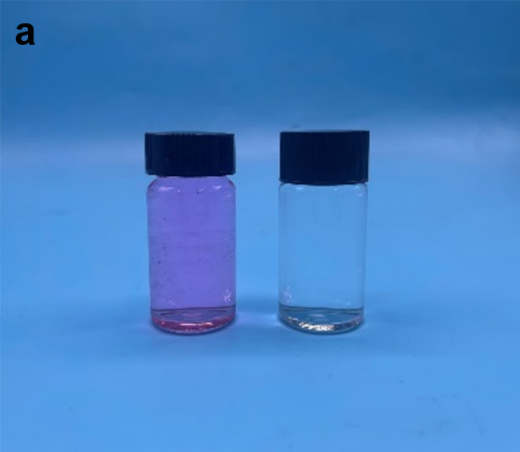

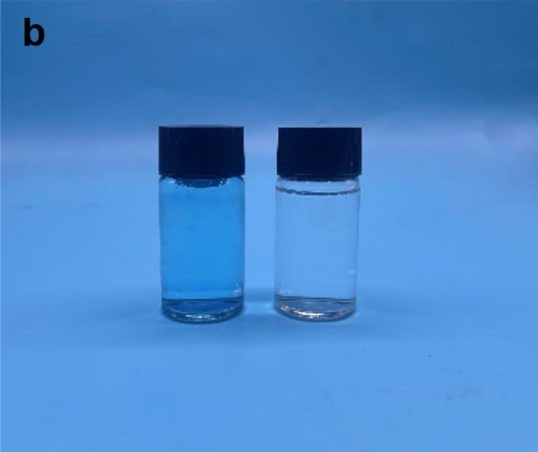


**Fig. S21 a** Digital photographs of the rhodamine B (RhB) aqueous solution before and after evaporation. **b** Digital photographs of the methylene blue (MB) aqueous solution before and after evaporation.


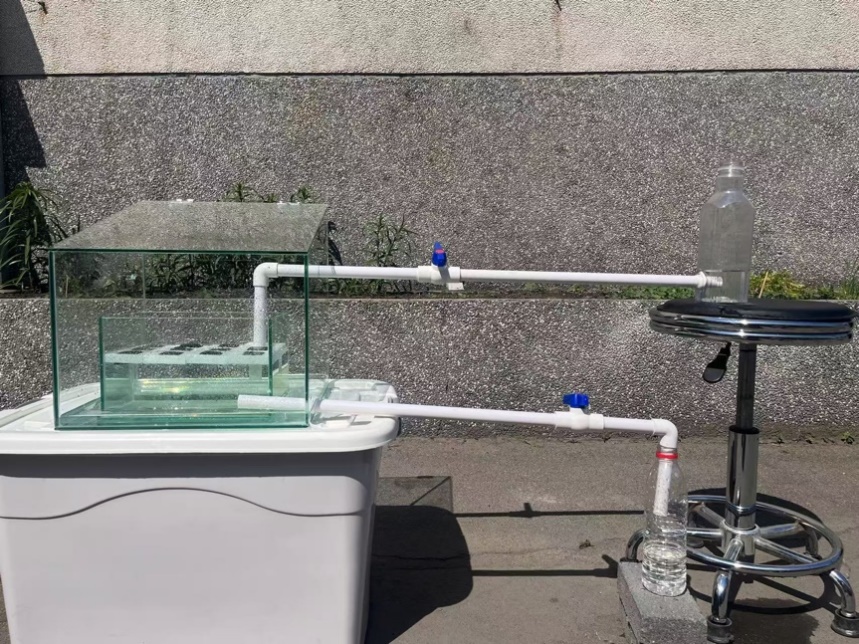


**Fig. S22** Diagram of the evaporator of the actual water collection device

**References**

1. W. Chong, R. Meng, Z. Liu, Q. Liu, J. Hu, B. Zhu, D. K. Macharia, Z. Chen, L. Zhang. Superhydrophilic polydopamine-modified carbon-fiber membrane with rapid seawater-transferring ability for constructing efficient hanging-model evaporator. *Adv. Fiber Mater.* 2023;5:1063-1075.

2. C. Niu, L. Yang, H. Sun, Z. Zhu, W. Liang, J. Li, A. Li. Three-dimensional macroporous foamy MXene membrane with high efficiency on interfacial evaporation and wastewater purification. *Chem. Eng. J.* 2023;476:Article 146522.

3. M. Xie, P. Zhang, Y. Cao, Y. Yan, Z. Wang, C. Jin. A three-dimensional antifungal wooden cone evaporator for highly efficient solar steam generation. *npj Clean Water.* 2023,6(1):12.

4. Y. Wu, R. Kong, C. Ma, L. Li, Y. Zheng, Y. Lu, L. Liang, Y. Pang, Q. Wu, Z. Shen, H. Chen. Simulation-guided design of bamboo leaf‐derived carbon-based high-efficiency evaporator for solar-driven interface water evaporation. *Energy Environ. Mater.* 2022;5(4):1323-1331.

5. H. Xing, Y. Song, H. Xu, S. Chen, K. Li, L. Dong, B. Wang, J. Xue, Y. Lu. A magneto-heated silk fibroin scaffold for anti-biofouling solar steam generation. *Small.* 2023;19(18): e2206189.

6. W. Li, T. Li, B. Deng, T. Xu, G. Wang, W. Hu, C. Si. Fabrication of a facile self-floating lignin-based carbon janus evaporators for efficient and stable solar desalination. *Adv. Compos. Hybrid Mater.* 2024;7:Article 52.

7. H. S. Kang, J. W. Zou, Y. Liu, L. Ma, J. R. Feng, et al., Synergistic effect of photothermal conversion in MXene/Au@Cu_2−x_S hybrids for efficient solar water evaporation. *Adv. Funct. Mater.* 2023;33(44):2303911.

8. Z. Lei, X. Sun, S. Zhu, K. Dong, X. Liu, L. Wang, X. Zhang, L. Qu, X. Zhang. Nature inspired mxene-decorated 3d honeycomb-fabric architectures toward efficient water desalination and salt harvesting. *Nano-Micro Lett.* 2021;14(1):10.

9. Q. Liang, D. Deng, Z. Xiao, C. Liu. A novel slide-like cotton-based evaporator with gradient evaporation strategy for seawater resource acquirement. *Chem. Eng. J*.2024;479: Article 147222.

10. W. Lu, D. Jiang, Z. Wang, X. Zhang, Q. Ding, Z. Zhang, X. Liu, L. Bai, Z. Li, Y. Liu. Simultaneous efficient evaporation and stable electricity generation enabled by a wooden evaporator based on composite photothermal effect. *Chem. Eng. J.*2024;496:Article:154361.

11. L. Hou, N. Wang, L.-J. Yu, J. Liu, S. Zhang, Z. Cui, S. Li, H. Li, X. Liu, L. Jiang, Y. Zhao. High-performance janus solar evaporator for water purification with broad spectrum absorption and ultralow heat loss. *ACS Energy Lett.* 2022;8(1):553-564.

12. X. Zhao, H. Zhang, K. Y. Chan, X. Huang, Y. Yang, X. Shen. Tree-inspired structurally graded aerogel with synergistic water, salt, and thermal transport for high-salinity solar-powered evaporation. *Nano-Micro Lett.* 2024;16(1):222.

13. Z. Wo, X. Sun, H. Sun, Y. Su, Y. Xie, N. Yang, X. Zhang. All-in-one design of wood evaporator with highly-efficient salt resistance for sustainable solar desalination and contaminated water purification. *Chem. Eng. J.* 2025;507: Article 160715.
